# Spatial+: A novel approach to spatial confounding

**DOI:** 10.1111/biom.13656

**Published:** 2022-03-30

**Authors:** Emiko Dupont, Simon N. Wood, Nicole H. Augustin

**Affiliations:** ^1^ Department of Mathematical Sciences University of Bath Bath UK; ^2^ School of Mathematics University of Edinburgh Edinburgh UK

**Keywords:** bias reduction, collinearity, forestry, partial thin plate spline regression, spatial confounding

## Abstract

In spatial regression models, collinearity between covariates and spatial effects can lead to significant bias in effect estimates. This problem, known as spatial confounding, is encountered modeling forestry data to assess the effect of temperature on tree health. Reliable inference is difficult as results depend on whether or not spatial effects are included in the model. We propose a novel approach, spatial+, for dealing with spatial confounding when the covariate of interest is spatially dependent but not fully determined by spatial location. Using a thin plate spline model formulation we see that, in this case, the bias in covariate effect estimates is a direct result of spatial smoothing. Spatial+ reduces the sensitivity of the estimates to smoothing by replacing the covariates by their residuals after spatial dependence has been regressed away. Through asymptotic analysis we show that spatial+ avoids the bias problems of the spatial model. This is also demonstrated in a simulation study. Spatial+ is straightforward to implement using existing software and, as the response variable is the same as that of the spatial model, standard model selection criteria can be used for comparisons. A major advantage of the method is also that it extends to models with non‐Gaussian response distributions. Finally, while our results are derived in a thin plate spline setting, the spatial+ methodology transfers easily to other spatial model formulations.

## 1 INTRODUCTION

Regression models for spatially referenced data use spatial random effects to account for residual spatial correlation in the response data. As first noted by Clayton *et al.* (1993), these models can be problematic when estimation of individual covariate effects are of interest. So‐called spatial confounding arises because spatial random effects may be correlated with spatially dependent covariates in the model and therefore interfere with their effect estimates. Reich *et al.* (2006) analyzed the issue using an example modeling the effect of socioeconomic status on stomach cancer incidence in the municipalities of Slovenia. When spatial effects are added to the model, the covariate effect disappears, suggesting the spatial effects have taken over a disproportionate part of the explanatory power. While in this example, the spatial effects take the form of an intrinsic conditional autoregressive (ICAR) random effect, spatial confounding is widely acknowledged as an issue that affects spatial models in general (see, eg, Hodges and Reich, 2010; Paciorek, 2010).

**FIGURE 1:**
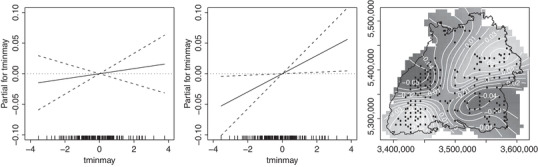
Forestry example. Estimated effect of minimum temperature in May on crown defoliation in the null model (left) and the spatial model (middle), where for each model the plot shows the contribution of the centered covariate to the predicted response (with two times standard error bands). Estimated spatial effect in the spatial model (right) with the border of Baden‐Württemberg outlined and dots showing the data locations

In this paper, we model data from the Terrestrial Crown Condition Inventory (TCCI) forest health monitoring survey which has been carried out yearly since 1983 by the Forest Research Institute Baden‐Württemberg. Crown defoliation (an indicator of poor tree health) has generally been worsening over time, and there is growing interest in understanding the effects of climate change in order to decide on forest management strategies for mitigation. Here, using a linear regression model, we consider the effect of temperature on crown defoliation. However, our results are highly dependent on whether or not we include spatial random effects in the model. As illustrated in Figure 1, in the null model (with no spatial effects), the estimated covariate effect is positive but not significant, whereas in the corresponding spatial model, the covariate effect is significant and the effect size more than triples. This behavior suggests there is spatial confounding and makes reliable inference difficult.

A commonly used method for dealing with spatial confounding is restricted spatial regression (RSR), introduced by Reich *et al.* (2006) for the ICAR model, and further developed by Hanks *et al.* (2015) for continuous space models. In RSR, the spatial random effects are restricted to the orthogonal complement of the covariates while keeping the overall column space of the model matrix in the regression unchanged. RSR directly eliminates collinearity and is designed to preserve the estimate of the null model while still accounting for residual spatial correlation. However, in the presence of unmeasured spatial confounders, the RSR estimate of the covariate effect may be significantly biased as it reflects not only the effect of the covariate but also that of the confounders (see, eg, Hanks *et al.*, 2015; Khan and Calder, 2020). Here, we define a spatial confounder in the classical sense of an unmeasured spatial variable (causal or otherwise) that is associated with both the covariate and the response (McNamee, 2003; Kirkwood and Sterne, 2010).

Paciorek (2010) and Page *et al.* (2017) study the behavior of the estimates in the spatial model when the covariate, like the response variable, has a spatial covariance structure. Intuitively, the model cannot distinguish the covariate from an unmeasured spatial effect, and the apportionment of effects between the covariate and spatial parts of the model may therefore be somewhat arbitrary. The analysis shows that the size of the resulting bias in the covariate effect estimate depends on the relative spatial scales of the covariate and spatial effects and, when the spatial scales agree, the bias is the same as that of RSR. Thus, while the estimate in the spatial model differs from RSR, it may be just as biased.

In many practical applications, however, the covariate of interest is spatially dependent but not fully determined by spatial location (ie, the covariate is not fully explained when a spatial model is fitted to it). This form of the covariate is assumed in Thaden and Kneib (2018) who propose the geoadditive structural equations model (gSEM). Here, spatial dependence is regressed away from both the response and the covariates, and a regression involving the residuals only is used to identify the original covariate effect. Simulations show that the bias in the covariate effect estimate of the spatial model is broadly removed using the gSEM. However, it is not immediately clear why the method works, and when the variables of interest are naturally spatially dependent, it seems undesirable to eliminate all spatial information from the modeling. The change in response variable also means that standard model selection criteria cannot be used for comparisons with the spatial and null models.

The structure of the covariate is usually not highlighted in the spatial confounding literature, but is important, as nonspatial information in a covariate can be used to distinguish it from the spatial effects without the need for considering differences in spatial scales. In this paper, we show that, when a covariate is not fully spatial (ie, not fully explained when a spatial model is fitted to it), unmeasured spatial confounders may still lead to significant bias in its effect estimate in the spatial model, however, the bias can be avoided in a relatively straightforward way. We propose a novel approach, spatial+, that is a simple modification of the spatial model in which the covariate is replaced by its residuals after spatial dependence has been regressed away. Similar to RSR, spatial+ retains the column space of the model matrix while reducing collinearity, but by adjusting the covariate rather than the spatial part of the model. Using asymptotic analysis as well as a simulation study we show that the estimates in spatial+ avoid the bias problems of the spatial model. We note that our asymptotic analysis applied to the gSEM estimates confirm the results of Thaden and Kneib (2018) and, for completeness, these derivations are included in Web Appendix D. An advantage of spatial+, however, is that all spatial information is retained in the model. Moreover, while the main properties of spatial+ are studied for models with a Gaussian response variable, we show that the method generalizes naturally to any response distribution from the exponential family of distributions.

Key to our analysis is that we formulate the spatial model as a partial thin plate spline model. Here, spatial correlation is modeled by imposing a smoothing penalty on the spatial effects in the fitting process. We then see that the bias in the covariate effect estimate arises as a direct result of smoothing, and spatial+ is a modification of the model matrix that makes the covariate part of the model less sensitive to this. Although our results are derived in the thin plate spline context, the methodology of spatial+ can be directly applied to other commonly used spatial models including, for example, Gaussian Markov random field (GMRF) models and the (discrete space) ICAR model. In fact, it can be shown that modeling spatial random effects through the use of a smoothing penalty is equivalent to a Bayesian model formulation in which the spatial correlation structure is determined by a prior distribution. This equivalence is explained, for example, in Kimeldorf and Wahba (1970), Silverman (1985, Section 6.1), Wood (2017, pp. 239–240), and Fahrmeir *et al.* (2004). Thus, while different spatial models correspond to different smoothing penalties, the underlying idea of reducing collinearity in this way to keep covariate effect estimates unaffected by spatial smoothing would apply in general.

This paper is structured as follows. In Section 2, we introduce the spatial and spatial+ models that form the basis of our analysis. Section 3 summarizes our asymptotic analysis, details of which are in the supplementary web material. In Section 4, we illustrate our theoretical results in a simulation study which also compares spatial+ with RSR and the gSEM. In Section 5, we demonstrate how spatial+ can be implemented by applying it to our forestry example. Finally, in Section 6, we generalize the spatial+ methodology to non‐Gaussian response distributions and confirm that the method works in simulations for three different distributions.

## 2 METHOD

### 2.1 Spatial model

Our starting point is a spatial model formulated as a partial thin plate spline model (see, eg, Wahba, 1990) of the form

(1) $$y_{i}=\beta x_{i}+f\left(t_{i}\right)+\varepsilon_{i},\;\varepsilon_{i}\overset{\mathrm{iid}}{∼}N\left(0,\sigma^{2}\right),$$

where $y={\left(y_{1},\text{…},y_{n}\right)}^{T}$ is the response, $x={\left(x_{1},\text{…},x_{n}\right)}^{T}$ an observed covariate with unknown effect β, and *f* an unknown bounded function defined on an open bounded domain $\Omega \subset R^{d}$ which includes the data locations $t_{1},\text{…},t_{n}$. The estimates $\hat{\beta }$ and $\hat{f}$ in this model (known as the partial thin plate spline estimates of order m>d/2) are obtained as the minimizers of

(2) $$\begin{array}{c} \frac{1}{n}\sum_{i=1}^{n}{\left(y_{i}-\beta x_{i}-f\left(t_{i}\right)\right)}^{2}+\lambda \sum_{i_{1},\text{…},i_{m}}\int_{R^{d}}|\frac{\partial^{m}f\left(t\right)}{\partial t_{i_{1}}\cdots \partial t_{i_{m}}}{|}^{2}dt, \end{array}$$

where λ>0 is an unknown smoothing parameter. Minimization here is over all β∈R and *f* is in a space of functions with square integrable partial derivatives of order *m* (for details, see Duchon, 1977). The first term encourages fitted values that are close to the data while the second term induces smoothing by penalizing the wiggliness of the function *f*.

Duchon (1977) showed that the estimate of *f* can be obtained by estimating its coefficients in a basis known as the natural thin plate spline basis. This basis spans a finite‐dimensional subspace in the space of functions defined on $R^{d}$ and has dimension N=M+n where M=m+d−1d. Using this basis, the partial thin plate spline estimates $\hat{\beta }$ and $\hat{f}={\left(\hat{f}\left(t_{1}\right),\text{…},\hat{f}\left(t_{n}\right)\right)}^{T}$ are the minimizers of

(3) $${∥y-\beta x-f∥}^{2}+n\lambda f^{T}\Gamma f$$

with Γ an n×n diagonal penalty matrix. Solving the resulting normal equations, we see that

(4) $$\hat{\beta }={\left(x^{T}\left(I-S_{\lambda }\right)x\right)}^{-1}x^{T}\left(I-S_{\lambda }\right)y,\;\hat{f}=S_{\lambda }\left(y-\hat{\beta }x\right),$$

where $S_{\lambda }={\left(I+n\lambda \Gamma \right)}^{-1}$ is known as the smoother matrix.

### 2.2 Spatial+ model

Starting with the model (1), in line with Rice (1986), we assume the covariate x has the form

(5) $$x_{i}=f^{x}\left(t_{i}\right)+\varepsilon_{i}^{x},\;\varepsilon_{i}^{x}\overset{\mathrm{iid}}{∼}N\left(0,\sigma_{x}^{2}\right),$$

where $f^{x}$ is a bounded smooth function defined on the spatial domain Ω. This means that x is correlated with the smooth term *f* through the component $f^{x}$. Extending the two‐stage smoothing spline model defined in Chen and Shiau (1991) to models of dimension d≥1, we define the spatial+ model as follows. Let ${\hat{f}}^{x}=S_{\lambda_{x}}x$ and $r^{x}=\left(I-S_{\lambda_{x}}\right)x$ be the fitted values and residuals in the thin plate spline regression (5) with smoothing parameter $\lambda_{x}>0$. The spatial+ model is then the partial thin plate spline model

(6) $$y_{i}=\beta r_{i}^{x}+f^{+}\left(t_{i}\right)+\varepsilon_{i},\;\varepsilon_{i}\overset{\mathrm{iid}}{∼}N\left(0,\sigma^{2}\right),$$

where β is the unknown effect of $r^{x}={\left(r_{1}^{x},\text{…},r_{n}^{x}\right)}^{T}$ and $f^{+}$ models the combined effect $f+\beta f^{x}$ in the original model (1). The spatial+ estimate ${\hat{\beta }}^{+}$ of β is its partial thin plate spline estimate in this model, that is,

(7) $$\begin{array}{ccc} {\hat{\beta }}^{+} & = & {\left(r^{xT}\left(I-S_{\lambda }\right)r^{x}\right)}^{-1}r^{xT}\left(I-S_{\lambda }\right)y\\ & = & {\left(x^{T}\left(I-S_{\lambda_{x}}\right)\left(I-S_{\lambda }\right)\left(I-S_{\lambda_{x}}\right)x\right)}^{-1}\;x^{T}\left(I-S_{\lambda_{x}}\right)\left(I-S_{\lambda }\right)y, \end{array}$$

and the spatial+ estimate ${\hat{f}}^{+}$ of $f={\left(f\left(t_{1}\right),\text{…},f\left(t_{n}\right)\right)}^{T}$ is given by

(8) $$\begin{array}{c} {\hat{f}}^{+}=S_{\lambda }\left(y-{\hat{\beta }}^{+}x\right)-{\hat{\beta }}^{+}{\hat{f}}^{x}=S_{\lambda }\left(y-{\hat{\beta }}^{+}x\right)-\left(I-S_{\lambda }\right)S_{\lambda_{x}}{\hat{\beta }}^{+}x. \end{array}$$

### 2.3 Smoothness selection

Smoothing penalties introduce bias in estimates but reduce variance. The smoothing parameters λ and $\lambda_{x}$ are usually estimated based on a separate smoothness selection criterion that balances this bias–variance trade‐off.

For the analysis summarized in Section 3, in line with Rice (1986) and Chen and Shiau (1991), we choose the value of the smoothing parameter that minimizes the average mean squared error (AMSE) of the estimated spatial effect. The AMSE for an estimated effect $\hat{f}={\left({\hat{f}}_{1},\text{…},{\hat{f}}_{n}\right)}^{T}$ of $f={\left(f\left(t_{1}\right),\text{…},f\left(t_{n}\right)\right)}^{T}$ is defined as

(9) $$\begin{array}{c} \mathrm{AMSE}\left(\hat{f}\right)=\frac{1}{n}\sum_{i=1}^{n}E\left[{\left({\hat{f}}_{i}-f\left(t_{i}\right)\right)}^{2}\right]=B^{2}\left(f,\lambda \right)+V\left(f,\lambda \right), \end{array}$$

where $B^{2}\left(f,\lambda \right)=\frac{1}{n}\sum_{i=1}^{n}{\left(E\left({\hat{f}}_{i}\right)-f\left(t_{i}\right)\right)}^{2}$ and $V\left(f,\lambda \right)=\frac{1}{n}\sum_{i=1}^{n}\mathrm{Var}\left({\hat{f}}_{i}\right)$ are the average squared bias and the average variance, respectively. (Note that $\hat{f}$ is a linear transformation of y and therefore has a multivariate normal distribution).

For the simulations in Section 4 and the example in Section 5 we use the generalized cross‐validation (GCV) criterion, which is the default option in the R‐package mgcv used for implementation. Asymptotically (as the sample size n→∞), GCV selects the optimal smoothing parameter for minimizing prediction error. Thus, GCV is not dissimilar to the criterion used for the theoretical derivations. Indeed, Chen and Shiau (1994) show that their asymptotic results in Chen and Shiau (1991) for one‐dimensional models also hold for GCV and Mallows' $C_{L}$. In practice, the restricted maximum likelihood (REML) criterion is often used instead of GCV as, for finite samples, GCV usually has more uncertain estimates than REML and tends to undersmooth (ie, overfit) the data (Wood, 2017, pp. 266–267). Repeating the simulations and the data example using REML gave similar results to GCV.

## 3 ASYMPTOTIC RESULTS

In the supplementary web material, we analyze the asymptotic behavior of effect estimates in the models defined in Sections 2.1 and 2.2 as the sample size n→∞. Without the smoothing penalty, the spatial model (1) is an ordinary linear model in which the estimates are unbiased. Therefore, bias in the covariate effect estimate $\hat{\beta }$ arises as a direct result of smoothing. In fact, for partial spline models (ie, models where the domain of the spline, here the spatial domain, is one‐dimensional) Rice (1986) identified this smoothing‐induced bias and showed that it can become disproportionately large unless the data are undersmoothed. More specifically, Rice's results show that if the smoothing parameter λ converges at the optimal rate (for minimizing the AMSE of the estimated smooth effect), it cannot be ensured that the bias of $\hat{\beta }$ converges faster than its standard deviation. Spatial+ is a higher‐dimensional version of a model introduced by Chen and Shiau (1991) to overcome this type of bias in dimension one.

Rice, Chen, and Shiau use the Demmler–Reinsch basis for natural splines to diagonalize the smoother matrix $S_{\lambda }$, enabling them to explicitly study the asymptotic behavior of the estimates in one‐dimensional models. Due to results by Utreras (1988), we are able to extend these derivations to models of arbitrary spatial dimension. The main results of our analysis are provided in Web Appendix C. We confirm that, as is the case in dimension one, when smoothing is chosen at an optimal rate for minimizing the AMSE of the estimated spatial effect, the bias in the covariate effect estimate in the spatial model can become disproportionately large. In contrast, in the spatial+ model, the bias converges to 0 strictly faster than the standard deviation. Our results are based on a number of technical lemmas and assumptions, details of which are provided in Web Appendices A and B.

## 4 SIMULATION

Partial thin plate spline models can be implemented in the R‐package mgcv using the computationally efficient reduced rank approximation known as thin plate regression splines. We use this implementation (with GCV as the smoothness selection criterion) to compare the results of models fitted to simulated data for which we know the true underlying covariate and spatial dependence.

### 4.1 Data

We generate 100 independent replicates of covariate data $x={\left(x_{1},\text{…},x_{n}\right)}^{T}$ and response data $y={\left(y_{1},\text{…},y_{n}\right)}^{T}$, observed at n=1000 randomly selected locations in the spatial domain [0, 10] × [0, 10] in $R^{2}$ (using a 50 × 50 grid), as follows. Let $z={\left(z_{1},\text{…},z_{n}\right)}^{T}$ and $z^{\prime }={\left(z_{1}^{\prime },\text{…},z_{n}^{\prime }\right)}^{T}$ denote observations at the selected locations of independently generated Gaussian spatial fields with an exponential and a spherical covariance structure, respectively. That is, each spatial field is sampled from a multivariate normal distribution centered at 0 with covariance structure defined by $C\left(h\right)=\exp(-{\left(h/R\right)}^{p})$ with R=5 and p=1 for the exponential field and $C\left(h\right)=-1-1.5h/R+0.5{\left(h/R\right)}^{3}$ for h≤R, C(h)=0 for h>R with R=1 for the spherical field (where *h* denotes Euclidean distance). To ensure that the fields lie in the span of the spatial basis vectors used for the models in Section 4.2, each is replaced by the fitted values of a spatial thin plate regression spline fitted to them. We then let

(10) $$\begin{array}{ccc} x & = & 0.5z+\varepsilon^{x}\mathrm{where}\varepsilon^{x}∼N\left(0,\sigma_{x}^{2}I\right),\\ y & = & \beta x+f+\varepsilon^{y}\mathrm{where}\varepsilon^{y}∼N\left(0,\sigma_{y}^{2}I\right), \end{array}$$

with true covariate effect β=3, true residual spatial effect $f=-z-z^{\prime }$, and $\sigma_{y}=1$, $\sigma_{x}=0.1$. Thus, f is directly correlated with the spatial pattern 0.5z of the covariate. This approach is similar to Thaden and Kneib (2018), except we have added the component $-z^{\prime }$ so that f could represent, for example, the combined effect of an unobserved covariate (with a similar spatial pattern to that of x) as well as an independent short‐range spatial process. Also, rather than treating the spatial fields as fixed, we generate new fields for each replicate in the simulation. This slight change in approach was chosen to show that results do not rely on any particular replicates of the spatial fields. We note that similar results were obtained when we repeated the simulations for a number of fixed spatial fields. Finally, we have chosen $\sigma_{x}$ relatively small (such that the model matrix for the spatial model has nearly collinear columns) and $\sigma_{y}$ relatively large (to encourage smoothing). This is the situation in which we would expect spatial confounding issues to arise which is also confirmed by the simulations in Thaden and Kneib (2018).

### 4.2 Models

To each replicate of simulated response data y and covariate data x, we fit the following models (with basis size k=300 for the thin plate regression splines). Models 2–5 are fitted twice: once with smoothing penalties applied (ie, where λ and $\lambda_{x}$ are estimated from the data) and once without (ie, where $\lambda =\lambda_{x}=0$). In mgcv, smoothing penalties are applied by default but can be removed using the option fx=TRUE. This option means that the smoothing parameter is fixed rather than estimated, defaulting to 0 (ie, no smoothing) when no value is specified.

1. Null model: The model with no spatial effects given by
(11) $$y_{i}=\beta x_{i}+\varepsilon_{i},\;\varepsilon ∼N\left(0,\sigma^{2}I\right),$$
  where β and σ^2^ are estimated parameters.
2. Spatial model: The model given by
(12) $$y_{i}=\beta x_{i}+f\left(t_{i}\right)+\varepsilon_{i},\;\varepsilon ∼N\left(0,\sigma^{2}I\right),$$
  where β and σ^2^ are estimated parameters and *f* a thin plate regression spline with $t_{1},\text{…},t_{n}$ the observed data locations.
3. RSR model: Let $B_{\mathrm{sp}}$ be the matrix whose columns are the spatial basis vectors in the model matrix from (12) (ie, the thin plate regression spline basis functions evaluated at the data locations) and let ${\tilde{B}}_{\mathrm{sp}}=\left(I-x{\left(x^{T}x\right)}^{-1}x^{T}\right)B_{\mathrm{sp}}$ be the projection of this onto the orthogonal complement of x. The RSR model is given by
(13) $$y_{i}=\beta x_{i}+{\tilde{f}}_{i}+\varepsilon_{i},\;\varepsilon ∼N\left(0,\sigma^{2}I\right),$$
  where β and σ^2^ are estimated parameters and $\tilde{f}={\left({\tilde{f}}_{1},\text{…},{\tilde{f}}_{n}\right)}^{T}$ is modeled the same way as the spatial effect in (12) but with $B_{\mathrm{sp}}$ replaced by ${\tilde{B}}_{\mathrm{sp}}$ in the model matrix.
4. gSEM: Let $r^{x}={\left(r_{1}^{x},\text{…},r_{n}^{x}\right)}^{T}$ and $r^{y}={\left(r_{1}^{y},\text{…},r_{n}^{y}\right)}^{T}$ denote the spatial residuals of x and y, that is, $r^{x}=x-{\hat{f}}^{x}$ where ${\hat{f}}^{x}$ are the fitted values in the regression
(14) $$x_{i}=f^{x}\left(t_{i}\right)+\varepsilon_{i}^{x},\;\varepsilon^{x}∼N\left(0,\sigma_{x}^{2}I\right),$$
  where $\sigma_{x}^{2}$ is estimated and $f^{x}$ a thin plate regression spline, and $r^{y}$ is the same but replacing x by y. The gSEM model is then the linear model given by
(15) $$r_{i}^{y}=\beta r_{i}^{x}+\varepsilon_{i},\;\varepsilon ∼N\left(0,\sigma^{2}I\right),$$
  where β and σ^2^ are estimated.
5. Spatial+: Let $r^{x}$ denote the spatial residuals of x as above. The spatial+ model is then
(16) $$y_{i}=\beta r_{i}^{x}+f^{+}\left(t_{i}\right)+\varepsilon_{i},\;\varepsilon ∼N\left(0,\sigma^{2}I\right),$$
  where β and σ^2^ are estimated parameters and $f^{+}$ a thin plate regression spline.

### 4.3 Results

**FIGURE 2:**
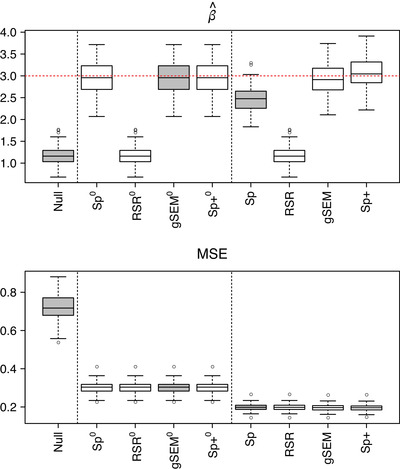
Estimated covariate effect $\hat{\beta }$ (top) and MSE of fitted values (bottom) for each model fitted to 100 data replicates, where the true covariate effect is β=3. Sp and Sp+ denote the spatial and spatial+ models, respectively, and superscript 0 refers to an unsmoothed model (ie, $\lambda =\lambda_{x}=0$). Results in gray are the three models that correspond to those used in Thaden and Kneib's simulation study. This figure appears in color in the electronic version of this article, and any mention of color refers to that version

The results of the simulation are summarized in Figure 2. For each data replicate, the output is the estimated covariate effect and the mean squared error (MSE) of fitted values for each model fit. For ease of notation, in this section, we use $\hat{\beta }$ to mean the estimated covariate effect in any of the fitted models (rather than the partial thin plate spline estimate alone). The MSE of fitted values is calculated as $∥\hat{y}{-\left(\beta x+f\right)∥}^{2}$ where for models 1, 2, 3, and 5, $\hat{y}$ is the fitted values in the regressions (11), (12), (13), and (16), respectively, and for model 4, $\hat{y}={\hat{f}}^{y}+{\hat{r}}^{y}$ where ${\hat{f}}^{y}$ and ${\hat{r}}^{y}$ are the fitted values in the regressions (14) and (15). Here, β=3 and $f=-z-z^{\prime }$ are the true values of the estimated effects with βx+f the true mean of y.

In the null model and the RSR model, the estimated covariate effect is the same (that is, for any given data replicate, the value of $\hat{\beta }$ is identical) and has a noticeably larger bias than the estimates in the other models. This is expected as for these models, $\hat{\beta }$ is the ordinary least squares estimate, which, in addition to the true effect β, includes a contribution from the part of f that is correlated with x. More specifically, the bias in $\hat{\beta }$ is given by $E({\left(x^{T}x\right)}^{-1}x^{T}f)$ (see Web Appendix E). Note that in our simulations, since x and f are negatively correlated, the bias is negative, however, if the correlation had been positive, the bias would have been positive. The fitted values in RSR, however, differ from those of the null model as the larger model matrix explains a part of y that is treated as random noise in the null model. In fact, the column space of the model matrix of the RSR model is the same as that of the spatial model, and it is therefore not surprising that, for any given data replicate, the fitted values in these two models are similar.

If no smoothing penalty is applied, models 2, 4, and 5 are essentially the same: for any given data replicate, they have the same fitted values and the same unbiased estimate for the covariate effect. This illustrates that spatial confounding bias is due to the combined effect of collinearity and smoothing, rather than collinearity alone. The spatial model is, in this case, an ordinary linear model where the columns in the model matrix are the covariate x and the spatial basis vectors $B_{\mathrm{sp}}$. This is the model from which the data are generated and, therefore, it is not surprising that the spatial model is able to recapture the true effects. The spatial+ model is a reparameterization of the spatial model which preserves the overall column space, and simple linear model theory shows that the covariate effect estimate is preserved. Similarly, it is straightforward to show that the gSEM covariate effect estimate agrees with the spatial model in this case. (Derivations are included in Web Appendix E.)

In the unsmoothed versions of models 2–5, for any given data replicate, the fitted values are the same across all models. When smoothing is applied, the MSE of fitted values reduces, indeed, this is the intended purpose of the smoothing penalty. Looking at the covariate effect estimate, in the RSR model, the (biased) ordinary least squares estimate is unaffected by smoothing. For the remaining three models, while the unsmoothed versions of the models give unbiased estimates of β, we see that smoothing introduces varying degrees of bias. In the spatial model, the bias is quite large illustrating our results from Section 3. In contrast, while the covariate effect estimate is no longer the same in the gSEM and the spatial+ model, for both models, the bias is still negligible. This behavior is therefore also consistent with what we would expect from our theoretical results.

### 4.4 Additional comments

Our analysis in Section 4.3 gives some intuition for why spatial+ works. If no smoothing penalty is applied we saw that for any given data replicate, spatial+ has the same unbiased estimate for the covariate effect as the spatial model. In fact, any decomposition x=v+r with v in the column space of the spatial basis vectors $B_{\mathrm{sp}}$ gives a reparameterization (replacing x by r) in which r captures the original covariate effect (since βx=βv+βr). However, by choosing r to be broadly orthogonal to the column space of $B_{\mathrm{sp}}$ (as it is in the spatial+ model), the estimates of the covariate and spatial effects are broadly decorrelated. Thus, the covariate effect estimate is largely unaffected when smoothing is applied to the spatial term and thereby remains broadly unbiased.

Although the asymptotic results in Section 3 are technically only expected to hold for large sample sizes *n*, the above intuition of spatial+ applies in general. In order to investigate the behavior at moderate sample sizes, we repeated the simulations for n=300, n=150, and n=50 (with spatial basis sizes $k_{\mathrm{sp}}=100$, $k_{\mathrm{sp}}=100$, and $k_{\mathrm{sp}}=30$, respectively). The results are included in Web Appendix F. We see that the smaller the sample size, the larger the variability of the estimate $\hat{\beta }$, particularly for the unsmoothed spatial model, gSEM and spatial+ model as well as the smoothed gSEM and spatial+ model. However, overall, the behavior of the bias looks broadly similar to the results for the larger sample size and is in line with the asymptotic results. We note that, when the spatial basis size *k*_sp_ is large compared to *n*, the MSE of fitted values in the unsmoothed models becomes relatively high. The thin plate regression spline basis is generally ordered with lower‐frequency spatial patterns first so that adding more spatial basis functions increases the ability of the spatial effect to model more complex spatial variation involving both lower‐ and higher‐frequency spatial patterns. In practice, while a small basis size is preferable for reducing computation time for model fitting, a large basis may be necessary in order to capture the spatial variation in the data. However, when the flexibility of the spatial effect is increased, the model is also more likely to overfit the data. The purpose of smoothing is exactly to avoid this overfitting and reduce the effective degrees of freedom in the model.

Finally, our results assume that the true spatial dependence of both the response variable and the covariate can be described by thin plate splines. (In our simulations, this was ensured by fitting thin plate splines to the spatial fields z and $z^{\prime }$ used for the data generation.) If this is not the case, there may be additional bias caused by model misspecification. In practical terms, the thin plate spline formulation assumes that spatial dependence is smooth and isotropic, but when these conditions hold it provides a fairly flexible way of modeling spatial effects. Thus, when we repeated our simulations using data based on spatial fields that were fitted with Gaussian process smooths rather than thin plate splines, the results were very similar. (See Web Appendix F).

## 5 APPLICATION

We illustrate how the spatial+ model can be used in practice by applying it to our forestry example. Details of the data can be found in Augustin *et al.* (2009) and Eichhorn *et al.* (2017). We consider here the data for spruce for a single observation year, namely, 2013 which has measurements from n=186 locations. We are interested in assessing the effect of the climate variable tminmay (minimum temperature in May) on the response variable ratio (crown defoliation expressed as a proportion). We expect a high minimum temperature in May to be indicative of a warmer and drier year in general which, in turn, is likely to lead to higher levels of tree defoliation (measured later in summer). We also expect older trees to have significantly more defoliation than younger trees and have therefore included the variable age (age of trees) as an additional covariate in the models. Scatterplots of the data (not shown here) indicate the relationships between the covariates and the response variable are broadly as expected.

### 5.1 Models

A natural starting point is the null model

(17) $${\mathrm{ratio}}_{i}=\alpha +\beta_{1}{\mathrm{age}}_{i}+\beta_{2}{\mathrm{tminmay}}_{i}+\varepsilon_{i},$$

where $\varepsilon_{i}∼N\left(0,\sigma^{2}\right)$ is iid noise and $\alpha ,\beta_{1},\beta_{2}$, and σ are estimated parameters. However, numerous spatially dependent predictors have not been included in the model, for example, soil characteristics such as soil depth and base saturation; other climatic variables such as those related to radiation and precipitation; water budget of the trees, and so forth. Therefore, we would expect residual spatial correlation in the response variable, and a more appropriate model may therefore be a spatial model, which we define as

(18) $${\mathrm{ratio}}_{i}=\alpha +\beta_{1}{\mathrm{age}}_{i}+\beta_{2}{\mathrm{tminmay}}_{i}+f\left(t_{i}\right)+\varepsilon_{i},$$

where $\varepsilon_{i}∼N\left(0,\sigma^{2}\right)$ is iid noise, $\alpha ,\beta_{1},\beta_{2}$, and σ are estimated parameters and *f* a thin plate regression spline (with basis size k=100) with $t_{1},\text{…},t_{n}$ the observed data locations.

The covariate effects of interest are β_1_ and β_2_ but, as the results of Sections 3 and 4 show, the estimates of these effects may be highly biased in both the null model and the spatial model. This disproportionate bias is avoided in the spatial+ model. Let $r^{1}={\left(r_{1}^{1},\text{…},r_{n}^{1}\right)}^{T}$ and $r^{2}={\left(r_{1}^{2},\text{…},r_{n}^{2}\right)}^{T}$ be the residuals when a thin plate regression spline (with basis size k=100) is fitted to age and tminmay, respectively. The spatial+ model is then

(19) $${\mathrm{ratio}}_{i}=\alpha +\beta_{1}r_{i}^{1}+\beta_{2}r_{i}^{2}+f^{+}\left(t_{i}\right)+\varepsilon_{i},$$

where $\varepsilon_{i}∼N\left(0,\sigma^{2}\right)$ is iid noise, $\alpha ,\beta_{1},\beta_{2}$, and σ are estimated parameters and $f^{+}$ a thin plate regression spline (with basis size k=100) with $t_{1},\text{…},t_{n}$ the observed data locations.

Finally, for comparison, we fit the gSEM as an alternative method for avoiding spatial confounding bias. Let $r^{y}={\left(r_{1}^{y},\text{…},r_{n}^{y}\right)}^{T}$ be the residuals when a thin plate regression spline (with basis size k=100) is fitted to the response variable ratio. The gSEM is then

(20) $$r_{i}^{y}=\beta_{1}r_{i}^{1}+\beta_{2}r_{i}^{2}+\varepsilon_{i},$$

where $\varepsilon_{i}∼N\left(0,\sigma^{2}\right)$ is iid noise and $\beta_{1},\beta_{2}$, and σ are estimated parameters.

### 5.2 Results

The results of fitting the above four models to the data are summarized in Table 1.

**TABLE 1:** Forestry example: results of fitting models to the data

	age			tminmay			s(x,y)					
	$\hat{\beta }$	*p*‐value		$\hat{\beta }$	*p*‐value		edf	*p*‐value		Dev expl	$\hat{\sigma }$	AIC
Null	0.00247	$<10^{-16}$	***	0.0042	0.5049					0.490	0.00940	−335
Spatial	0.00237	$<10^{-16}$	***	0.0149	0.0307	*	14.2	0.0243	*	0.605	0.00789	−355
Spatial+	0.00237	$<10^{-16}$	***	0.0316	0.0073	**	12.0	3.32e−05	***	0.598	0.00793	−356
gSEM	0.00232	$<10^{-16}$	***	0.0317	0.0058	**						

*Note*: For each covariate: the estimate of the covariate effect β and its *p*‐value. s(x,y) refers to the thin plate regression splines fitted to *f* in the spatial model and $f^{+}$ in the spatial+ model. For each of these: the effective degrees of freedom (edf) and the *p*‐value. For each significant *p*‐value, we write *** if it is <0.001, ** if <0.01, and * if <0.05. Note that in the gSEM, deviance explained, estimated standard deviation, and AIC do not compare directly with the other models as the response variable is different.

The spatial term in the spatial model is significant, which confirms there is residual spatial correlation in the data as expected. Furthermore, as the spatial term allows for more of the residual variation to be explained, the deviance explained is higher and the estimated standard deviation is lower than in the null model. As the Akaike information criterion (AIC) is also lower, we conclude that the spatial model is an overall better fitting model than the null model for this data. However, while the spatial model may be appropriate for overall predictions of the response variable, the estimate of any individual covariate effect may be biased. Using the spatial+ model, we expect to obtain similar fitted values as the spatial model but with covariate effect estimates that have only negligible bias. Indeed, in terms of overall fit, we see that the deviance explained, estimated standard deviation, and AIC in the spatial+ model are similar to those of the spatial model. For completeness, we have also included the gSEM. Note, however, that in the gSEM, since the response variable in the regression differs from that of the other three models, the deviance explained, estimated standard deviation, and AIC cannot be directly compared to the other models.

The covariate age is highly significant and has a positive effect as expected. This covariate does not appear to be affected by spatial confounding as the estimated effect and its *p*‐value are largely robust to the choice of model. This happens, for example, if a covariate is independent of the true underlying residual spatial effect. Also, in the case of age, not only is this a covariate that is not very well explained by spatial location (a spatial smooth fitted to this variable has deviance explained of only 13%), but its estimated spatial pattern looks dominated by linear spatial basis functions which are unpenalized in the spatial model. Therefore, penalization of the spatial term *f* in the spatial model is less likely to interfere with the covariate effect estimate (see Rice, 1986, Proposition D).

In contrast, the estimated effect of the covariate tminmay is not significant in the null model but is significant in the spatial model and is even more significant in the spatial+ model. Furthermore, while in all models the effect estimate is positive as expected (ie, higher temperature in May leads to more defoliation later in summer), the size of the estimate more than triples when a spatial effect is added to the null model and the estimate in the spatial+ model is more than double that in the spatial model. This shows that, if we were to use the spatial model for our inference, the effect of temperature on crown defoliation would likely be underestimated in both size and significance due to spatial confounding. Note that, as expected, the gSEM gives similar results to spatial+.

## 6 NON‐GAUSSIAN RESPONSE DATA

In this section, we generalize the models from Section 2 to response distributions from the exponential family, which includes, for example, the Poisson, gamma, and binomial distributions.

### 6.1 Spatial model

Suppose we have response data $y={\left(y_{1},\text{…},y_{n}\right)}^{T}$ where each $y_{i}$ is assumed to be a random variable whose distribution is from the exponential family with $E\left(y_{i}\right)=\mu_{i}$, and suppose $x={\left(x_{1},\text{…},x_{n}\right)}^{T}$ and $t_{1},\text{…},t_{n}$ are covariate observations and spatial locations as before. A generalized version of (1) can then be formulated as

(21) $$g\left(\mu_{i}\right)=\beta x_{i}+f\left(t_{i}\right),$$

where β is an unknown parameter, *f* a thin plate spline, and g:R→R a link function (ie, a monotonic smooth function which ensures $g(\mu_{i})$ is in the domain of the response variable). The partial thin plate spline estimates $\hat{\beta }$ and $\hat{f}={\left(\hat{f}\left(t_{1}\right),\text{…},\hat{f}\left(t_{n}\right)\right)}^{T}$ are found using the penalized iterative re‐weighted least squares (PIRLS) algorithm (for details, see Web Appendix G). If no smoothing is applied, these estimates are the maximum likelihood estimates in a generalized linear model (GLM), which are asymptotically unbiased.

### 6.2 Spatial+ model

Starting with the model (21), let W and z denote the weights matrix and pseudodata at convergence of the PIRLS algorithm (described in Web Appendix G). We then define the corresponding spatial+ model as follows. Let ${\hat{f}}^{x}$ and $r^{x}=x-{\hat{f}}^{x}={\left(r_{1}^{x},\text{…},r_{n}^{x}\right)}^{T}$ denote the fitted values and residuals in the weighted thin plate regression (5) with weights W, that is, ${\hat{f}}^{x}$ is the minimizer of $∥\sqrt{W}\left(x-f^{x}\right){∥}^{2}+n\lambda_{x}f^{xT}\Gamma f^{x}$ with smoothing parameter $\lambda_{x}>0$ and Γ defined as in (3). The spatial+ model is then the partial thin plate spline model defined by

(22) $$g\left(\mu_{i}\right)=\beta r_{i}^{x}+f^{+}\left(t_{i}\right),$$

where β and $f^{+}$ are estimated as in Section 6.1. For further details, see Web Appendix G.

### 6.3 Simulations

The models (21) and (22) can once again be implemented using thin plate regression splines in mgcv. To test the performance of the spatial+ model (22), we repeat the simulations from Section 4 for three different response distributions, namely, the Poisson distribution with canonical link function g(μ)=log(μ), the exponential distribution with (noncanonical) link function g(μ)=log(μ), and the binomial distribution with size parameter $n_{\mathrm{bin}}=10$ and canonical link function $g\left(\mu \right)=\log(\mu /\left(n_{\mathrm{bin}}-\mu \right))$.

**FIGURE 3:**
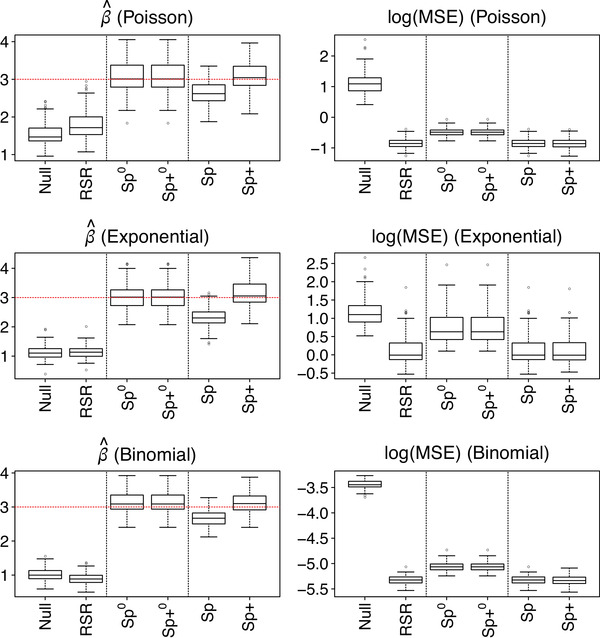
For each of the distributions Poisson (top), exponential (middle), binomial (bottom): the estimated covariate effect $\hat{\beta }$ (left) and log(MSE) of fitted values (right) for each model fitted to 100 data replicates, where the true covariate effect is β=3. Sp and Sp+ denote the spatial and spatial+ models, respectively, and superscript 0 refers to an unsmoothed model (ie, $\lambda =\lambda_{x}=0$). This figure appears in color in the electronic version of this article, and any mention of color refers to that version

For each response distribution, we simulate 100 replicates of the response data $y={\left(y_{1},\text{…},y_{n}\right)}^{T}$ by independently sampling each $y_{i}$ from the given distribution with mean $\mu_{i}=g^{-1}\left(\eta_{i}\right)$ where $\eta_{i}=\beta x_{i}+f_{i}$ with $x={\left(x_{1},\text{…},x_{n}\right)}^{T}$ simulated as in Section 4.1, $\sigma_{x}=0.1$, and true effects β=3, $f={\left(f_{1},\text{…},f_{n}\right)}^{T}=-z-z^{\prime }$ as before. The results of fitting the models (21) and (22) are summarized in Figure 3. For comparison, we have also included the results of fitting the corresponding null model (ie, the GLM defined by $g\left(\mu_{i}\right)=\beta x_{i}$) and the models (21) and (22) with no smoothing penalty applied. Finally, we have fitted a generalized version of the RSR model (for details, see Web Appendix G). Note that we have not included the gSEM here as it is not immediately clear how to generalize this model to non‐Gaussian response distributions.

We see that for all three response distributions, the overall behavior of the models is similar to what we saw in the Gaussian case. As before, the null model and RSR model both have highly biased covariate effect estimates; however, note that unlike the Gaussian case, the estimate is not the same in the two models. This is because, while in both models the estimate is given by $\hat{\beta }={\left(x^{T}Wx\right)}^{-1}x^{T}Wz$, the fitted values, and hence the weights and pseudodata at convergence, differ. Without smoothing, as expected, the spatial and spatial+ models give the same results, however, while the covariate effect estimate looks unbiased for the Poisson and exponential response distributions, it looks slightly biased for the binomial distribution, though not materially. This is not surprising as GLMs are only asymptotically unbiased and may have some bias in practice, particularly, when the number of estimated parameters is relatively large as it is in this case (Cox and Snell, 1968). When smoothing is applied, MSE reduces as intended, but the covariate effect estimate in the spatial model becomes significantly biased while it remains broadly unbiased in the spatial+ model.

## 7 DISCUSSION

We have shown that for covariates that are spatially dependent but not fully spatial (ie, each covariate is not fully explained when a spatial model is fitted to it), the proposed spatial+ model can be used to avoid unreliable effect estimates in spatial regression with clear advantages over existing methods. Our analysis also gives a clearer understanding of spatial confounding in this context. Spatial models, whether formulated in terms of spatially induced prior distributions or smoothing penalties, usually apply some form of spatial smoothing to reflect spatial correlation in the data and avoid overfitting. However, from the model formulation (1), we see that it is exactly this smoothing that causes spatial confounding bias as, without smoothing, the spatial model has unbiased estimates. The nonspatial information in the covariate means that the model can distinguish it from an unmeasured spatial confounder. However, if the correlation between the covariate and the spatial confounder is high, the smoothing applied to the spatial term in the model can disproportionately affect the estimate of the covariate effect.

The excessive smoothing‐induced bias is avoided in both spatial+ and the gSEM. If no smoothing penalty is applied, both models give the same unbiased covariate effect estimates as the unsmoothed spatial model. Spatial+ reparameterizes the spatial model so that spatial dependence is removed from the covariates and instead fully contained in the spatial term $f^{+}$. This makes fixed‐effect estimates broadly independent of the spatial effects, in particular, they remain largely unbiased under spatial smoothing. The idea of decorrelating covariate and spatial terms is also used in RSR, however, restricting the spatial effects leads to bias by construction. In the gSEM, the elimination of all spatial information means that fixed‐effect estimates are once again decorrelated from the spatial effects and thereby protected from spatial smoothing. The resulting model of residuals only, however, seems less intuitive than spatial+ and, the change in response variable means that standard model selection criteria cannot be used for comparisons with the other models. A major advantage of spatial+ is also that the method generalizes easily to models with non‐Gaussian response distributions and our simulations illustrate that the method still works well here.

Our above discussion shows that the decorrelation of effect estimates is the underlying reason why the spatial+ approach works. As mentioned in Section 1, the modification of the model matrix that achieves this is easily transferable to other spatial model formulations, and we would therefore expect the method to work well in general. However, as our theoretical derivations are specific to thin plate spline estimates, similar derivations or simulations could be done to confirm our results in other settings. One limitation to the spatial+ approach is that the covariate effects in the model must be linear. This assumption is needed for the spatial residuals to capture the true covariate effects. The spatial model (1) is easily extended, using the generalized additive model (GAM) framework, to include nonlinear covariate terms in the form of smooths (ie, unknown functions of the covariates estimated from the data). It would be interesting to see if any of the ideas of spatial+, as well as our increased understanding of spatial confounding, can be used to develop methods for avoiding spatial confounding in this context.

Finally, applying spatial+ to the forestry example, we see that the effect of temperature on crown defoliation appears to be positive and significant as expected, and that this effect would likely be underestimated in both size and significance in the spatial model (and even more so in the null model). The other covariate, age of trees, in this example also illustrates that, if a covariate is not spatially confounded, this can be confirmed by showing that its effect estimate in the spatial and spatial+ models agree. It is possible that this idea could be used to develop a diagnostic or test that practitioners could use to identify spatial confounding in applications.

## Supporting information

Web Appendices referenced in Sections 1, 3, 4 and 6, along with the R code for Sections 4, 5 and 6 are available with this paper at the Biometrics website on Wiley Online Library.

Supporting Information

## Data Availability

The data that support the findings in this paper are available in the Supporting Information of this article.
