# Supplementary material for: Spatial+: A novel approach to spatial confounding
Source: Biometrics. 2022 Mar 30;78(4):1279–90. doi: 10.1111/biom.13656 (PMC10084199; doi:10.1111/biom.13656)
Supplement: Supplementary file 1 — Web Appendices referenced in Sections 1, 3, 4 and 6, along with the R code for Sections 4, 5 and 6 are available with this paper at the Biometrics website on Wiley Online Library. [file BIOM-78-1279-s002.pdf]

Supporting information for "Spatial+: a novel approach to spatial confounding" by Emiko Dupont, Simon N. Wood and Nicole H. Augustin

1 Web Appendix A: Technical lemmas

In this appendix we set out the technical lemmas that we use for the derivations of the main results of our asymptotic analysis (detailed in Web Appendix C below), which generalize the results of Rice (1986); Chen and Shiau (1991) from $d = 1$ to dimensions $d \geq 1$. Key to this generalization is the following result by Utreras (1988) on the asymptotics of thin plate splines.

Lemma 1.1. *Suppose Ω has Lipschitz boundary and satisfies a uniform cone condition (as defined in Utreras (1988)). Assume that the points $\{\mathbf{t}_1, \ldots, \mathbf{t}_n\} \subset \Omega$ are regularly distributed in the sense that there exists a constant $B > 0$ such that*

$$\frac{h_{\min}}{h_{\max}} \leq B$$

where $h_{\max} = \sup_{\mathbf{t} \in \Omega} \inf_i |\mathbf{t} - \mathbf{t}_i|$ and $h_{\min} = \min_{i \neq j} |\mathbf{t}_i - \mathbf{t}_j|$. Let $\mu_1 \leq \cdots \leq \mu_n$ denote the eigenvalues of the matrix $n\boldsymbol{\Gamma}$ and assume $m > d/2$. Then

$$\mu_1 = \cdots = \mu_M = 0$$

and there exist constants $C_1, C_2 > 0$ such that

$$C_1 k^{2m/d} \leq \mu_k \leq C_2 k^{2m/d} \quad \text{for } M + 1 \leq k \leq n.$$

Proof. See the proof of Theorem 5.1 (a) and Theorem 5.3 of Utreras (1988). □

Lemma 1.1 provides us with a convenient basis in which the smoother matrix $\mathbf{S}_\lambda = (\mathbf{I} + n\lambda\boldsymbol{\Gamma})^{-1}$ is diagonalized and, moreover, describes the asymptotic behaviour of its eigenvalues as the number of data points $n \to \infty$. More specifically, if $\boldsymbol{\Phi}$ is the matrix whose columns are $\frac{1}{\sqrt{n}}\boldsymbol{\phi}_1, \ldots, \frac{1}{\sqrt{n}}\boldsymbol{\phi}_n$ where $\boldsymbol{\phi}_k$ is an eigenvector of $n\Gamma$ corresponding to the eigenvalue μ_k, then (with appropriate scaling of the eigenvectors) $\boldsymbol{\Phi}$ has orthonormal columns and

$$\begin{aligned} \boldsymbol{\Phi}^T \mathbf{S}_\lambda \boldsymbol{\Phi} &= \operatorname{diag}\big(1/(1+\lambda\mu_1), \ldots, 1/(1+\lambda\mu_n)\big), \\ \boldsymbol{\Phi}^T (\mathbf{I} - \mathbf{S}_\lambda) \boldsymbol{\Phi} &= \operatorname{diag}\big((\lambda\mu_1)/(1+\lambda\mu_1), \ldots, (\lambda\mu_n)/(1+\lambda\mu_n)\big). \end{aligned}$$

This representation allows us to explicitly evaluate the estimates in the models of dimension $d \geq 1$ which, in turn, enables us to obtain asymptotic results in a similar way to Rice (1986); Chen and Shiau (1991).

For the rest of the supplementary web material, we assume that $m > d/2$ and that the domain Ω and the data points $\mathbf{t}_1, \ldots, \mathbf{t}_n$ satisfy the conditions of Lemma 1.1. We will also use the notation $a(n) \approx b(n)$ to mean that $a(n)/b(n)$ is bounded away from zero and infinity as $n \to \infty$.

Lemmas 1.2 and 1.3 link the asymptotic behaviour of the smoother matrix $\mathbf{S}_\lambda$ to the convergence rate of the smoothing parameter λ. Lemma 1.2 generalizes Lemma 2 of Chen and Shiau (1991) to dimensions $d \geq 1$, and is proved using the asymptotic properties of the eigenvalues given in Lemma 1.1. The result in

Lemma 1.3 is proved by Utreras (1988). Lemmas 1.4 and 1.5 prove a number of asymptotic results that are convenient for later proofs. Lemma 1.4 shows how the results used by Rice (1986) for the analysis in dimension $d = 1$ generalize to dimensions $d \geq 1$, while Lemma 1.5 generalizes Lemma 3 of Chen and Shiau (1991) to dimensions $d \geq 1$. Proofs of Lemmas 1.2, 1.4 and 1.5 are given in Web Appendix B.

Lemma 1.2. *Suppose* $\lambda \approx n^{-\delta}$ *for some* $0 < \delta < 1$. *Then*

(a) $Tr(\mathbf{S}_\lambda) = \sum_{k=1}^{n}(1 + \lambda\mu_k)^{-1} = M + \mathcal{O}(\lambda^{-d/2m})$,

(b) $Tr(\mathbf{S}_\lambda^2) = \sum_{k=1}^{n}(1 + \lambda\mu_k)^{-2} = M + \mathcal{O}(\lambda^{-d/2m})$.

In particular, if $m \geq d$, *then both of these sums are of the form* $\mathcal{O}(n^{1/2-\tau})$ *where* $0 < \tau < 1/2$ *depends only on* δ.

Proof. See Web Appendix B. □

Lemma 1.3. *For any* $g \in H^m(\Omega)$, *let* $\mathbf{g} = (g(\mathbf{t}_1), \ldots, g(\mathbf{t}_n))^T$. *The averaged squared bias* $B_{\text{tp}}^2(g, \lambda)$ *of the thin plate spline* $\mathbf{S}_\lambda \mathbf{g}$ *(i.e. the fitted values in a model of the form (1) in our paper with* $\beta = 0$*) is given by*

$$B_{\text{tp}}^2(g, \lambda) = \frac{1}{n}\mathbf{g}^T(\mathbf{I} - \mathbf{S}_\lambda)^2\mathbf{g} = \mathcal{O}(\lambda).$$

Proof. See Utreras (1988) Lemma 2.2. □

Lemma 1.4. *Suppose* $\lambda \approx n^{-\delta}$ *for some* $0 < \delta < 1$, $f, f^x \in H^m(\Omega)$ *are bounded and* $m \geq d$. *Let* $\mathbf{f} = (f(\mathbf{t}_1), \ldots, f(\mathbf{t}_n))^T$. *Then*

(a) $n^{-1}\mathbf{x}^T(\mathbf{I} - \mathbf{S}_\lambda)\mathbf{x} = \sigma_x^2 + o(1)$,

(b) $n^{-1}\mathbf{x}^T(\mathbf{I} - \mathbf{S}_\lambda)^2\mathbf{x} = \sigma_x^2 + o(1)$,

(c) $n^{-1}\mathbf{x}^T(\mathbf{I} - \mathbf{S}_\lambda)\mathbf{f} = o(n^{-1/2}) + \mathcal{O}(\lambda^{-1/2})$,

(d) $n^{-1}\mathbf{x}^T\mathbf{S}_\lambda^2\mathbf{x} = \mathcal{O}(1)$

Proof. See Web Appendix B. □

Lemma 1.5. *Suppose* $\lambda \approx n^{-\delta}, \lambda_x \approx n^{-\delta_x}$ *for some* $0 < \delta, \delta_x < 1$, $f, f^x \in H^m(\Omega)$ *and* $m \geq d$. *Let* $\mathbf{f} = (f(\mathbf{t}_1), \ldots, f(\mathbf{t}_n))^T$. *Then*

(a) $n^{-1}\mathbf{x}^T(\mathbf{I} - \mathbf{S}_{\lambda_x})(\mathbf{I} - \mathbf{S}_\lambda)(\mathbf{I} - \mathbf{S}_{\lambda_x})\mathbf{x} = \sigma_x^2 + o(1)$,

(b) $n^{-1}\mathbf{x}^T(\mathbf{I} - \mathbf{S}_{\lambda_x})(\mathbf{I} - \mathbf{S}_\lambda)^2(\mathbf{I} - \mathbf{S}_{\lambda_x})\mathbf{x} = \sigma_x^2 + o(1)$,

(c) $n^{-1}\mathbf{x}^T(\mathbf{I} - \mathbf{S}_{\lambda_x})(\mathbf{I} - \mathbf{S}_\lambda)\mathbf{f} = o(n^{-1/2}) + \mathcal{O}((\lambda_x\lambda)^{1/2})$,

(d) $n^{-1}\mathbf{x}^T(\mathbf{I} - \mathbf{S}_{\lambda_x})(\mathbf{I} - \mathbf{S}_\lambda)\mathbf{S}_{\lambda_x}\mathbf{x} = o(n^{-1/2}) + \mathcal{O}((\lambda_x\lambda)^{1/2})$,

(e) $n^{-1}\mathbf{x}^T\mathbf{S}_{\lambda_x}(\mathbf{I} - \mathbf{S}_\lambda)^2\mathbf{S}_{\lambda_x}\mathbf{x} = \mathcal{O}(\lambda) + \mathcal{O}(n^{-1}\lambda_x^{-d/2m}\log^2 n)$

(f) $n^{-1}\mathbf{x}^T[\mathbf{S}_\lambda + (\mathbf{I} - \mathbf{S}_\lambda)\mathbf{S}_{\lambda_x}]^T[\mathbf{S}_\lambda + (\mathbf{I} - \mathbf{S}_\lambda)\mathbf{S}_{\lambda_x}]\mathbf{x} = \mathcal{O}(1)$,

Proof. See Web Appendix B. □

2 Web Appendix B: Proofs of technical lemmas

In this appendix we prove the lemmas set out in Web Appendix A. We start by introducing some notation. Recall the assumption from our paper that

$$x_i = f^x(\mathbf{t}_i) + \epsilon_i^x, \quad \epsilon_i^x \sim_{\text{iid}} N(0, \sigma_x^2)$$

which means that the covariate $\mathbf{x}$ is correlated with the smooth f in the spatial model. Therefore, $\mathbf{x}$ decomposes as

$$\mathbf{x} = \mathbf{f}^x + \boldsymbol{\epsilon}^x \tag{1}$$

with $\mathbf{f}^x = (f^x(\mathbf{t}_1), \dots, f^x(\mathbf{t}_n))^T$ and $\boldsymbol{\epsilon}^x = (\epsilon_1^x, \dots \epsilon_n^x)^T$. For the asymptotic analysis, it is often convenient to consider the behaviour of the components in this decomposition separately. Let $\mathbf{c}^x = (c_1^x, \dots, c_n^x)^T$ and $\boldsymbol{\xi}^x = (\xi_1^x, \dots, \xi_n^x)^T$ denote the coefficients of $\mathbf{f}^x$ and $\boldsymbol{\epsilon}^x$, respectively, in the basis $\boldsymbol{\Phi}$ introduced in Web Appendix A, i.e.

$$\begin{aligned} \mathbf{f}^x &= \boldsymbol{\Phi}\mathbf{c}^x \quad \text{where } \mathbf{c}^x = \boldsymbol{\Phi}^T\mathbf{f}^x, \\ \boldsymbol{\epsilon}^x &= \boldsymbol{\Phi}\boldsymbol{\xi}^x \quad \text{where } \boldsymbol{\xi}^x = \boldsymbol{\Phi}^T\boldsymbol{\epsilon}^x. \end{aligned}$$

Note that since $f^x \in H^m(\Omega)$ is bounded, we have that

$$n^{-1}\sum_{k=1}^{n}(c_k^x)^2 = n^{-1}(\mathbf{f}^x)^T(\mathbf{f}^x) \to 0 \text{ as } n \to \infty. \tag{2}$$

As in Rice (1986) and Chen and Shiau (1991), we also note that the following assumptions hold for the coefficients $\boldsymbol{\xi}^x$ of the iid noise $\boldsymbol{\epsilon}^x$.

(A1) $n^{-1}\sum_{k=1}^{n}\xi_k^x \to 0$ as $n \to \infty$,

(A2) $n^{-1}\sum_{k=1}^{n}(\xi_k^x)^2 = n^{-1}(\boldsymbol{\epsilon}^x)^T\boldsymbol{\epsilon}^x \to \sigma_x^2 > 0$ as $n \to \infty$,

(A3) $\sup_{1\le k\le n}|\xi_k^x| = \mathcal{O}(\log n)$.

Proof of Lemma 1.2

From Lemma 1.1, $\mu_k = 0$ for $k = 1, \dots, M$, so $\sum_{k=1}^{M}(1+\lambda\mu_k)^{-1} = M$. Split the remaining range of the summation into $I_1 = [M+1, \lambda^{-d/2m}]$, $I_2 = [\lambda^{-d/2m}, n]$.

I_1: Since $(1+\lambda\mu_k)^{-1} \le 1$ for all k

$$\sum_{I_1}(1+\lambda\mu_k)^{-1} \le \sum_{I_1} 1 \le \lambda^{-d/2m}.$$

I_2: By Lemma 1.1, $(1+\lambda\mu_k)^{-1} \le (C_1\lambda k^{2m/d})^{-1}$ for all k in I_2. Since $\{\mu_k\}_k$ is an increasing sequence, we have that

$$\begin{aligned} \sum_{I_2}(1+\lambda\mu_k)^{-1} &\le \int_{\lambda^{-d/2m}}^{\infty}(C_1\lambda x^{2m/d})^{-1}dx \\ &= C\lambda^{-d/2m} \end{aligned}$$

where $C = (C_1(2m/d-1))^{-1}$. This proves part (a).

For part (b) we note that $\sum_{k=1}^{M}(1+\lambda\mu_k)^{-2} = M$ as before and that $(1+\lambda\mu_k)^{-2} < (1+\lambda\mu_k)^{-1}$ for all the remaining k. Therefore (b) follows from (a).

Proof of Lemma 1.4

To prove (a), we use the decomposition $\mathbf{x} = \mathbf{f}^x + \boldsymbol{\epsilon}^x$ from (1) and the corresponding basis expansions in the basis $\boldsymbol{\Phi}$ to get

$$n^{-1}\mathbf{x}^T(\mathbf{I} - \mathbf{S}_\lambda)\mathbf{x} = n^{-1}\sum_k (c_k^x + \xi_k^x)^2 \frac{\lambda\mu_k}{1+\lambda\mu_k}.$$

We note that while

$$(c_k^x + \xi_k^x)^2 = (c_k^x)^2 + (\xi_k^x)^2 + 2c_k^x\xi_k^x,$$

due to the Cauchy-Schwarz inequality, the term $2c_k^x\xi_k^x$ will never dominate the rate of convergence. Therefore, we only need to consider the parts of the sum relating to the other two terms. Using Cauchy-Schwarz again we see that

$$\begin{aligned}
\sum_k (c_k^x)^2 \frac{\lambda\mu_k}{1+\lambda\mu_k} &\leq \Big(\sum_k (c_k^x)^2 \big(\frac{\lambda\mu_k}{1+\lambda\mu_k}\big)^2\Big)^{1/2}\Big(\sum_k (c_k^x)^2\Big)^{1/2} \\
&= \big(nB_{\text{tp}}^2(f^x,\lambda)\big)^{1/2}\big(\sum_k (c_k^x)^2\big)^{1/2} \\
&= \mathcal{O}(n\lambda^{1/2}) = \mathcal{O}(n^{1-\delta/2}) = o(n).
\end{aligned}$$

Here we have used Lemma 1.3 and (2).

For the term involving $(\xi_k^x)^2$ we have that

$$\begin{aligned}
\sum_k (\xi_k^x)^2 - \sum_k (\xi_k^x)^2 \frac{\lambda\mu_k}{1+\lambda\mu_k} &= \sum_k (\xi_k^x)^2 \frac{1}{1+\lambda\mu_k} \\
&\leq \sup_k (\xi_k^x)^2 \sum_k \frac{1}{1+\lambda\mu_k} \\
&= \mathcal{O}(\log^2 n)\mathcal{O}(n^{1/2-\tau}) = o(n)
\end{aligned}$$

by assumption (A3) and Lemma 1.2. Hence, by assumption (A2),

$$n^{-1}\sum_k (\xi_k^x)^2 \frac{\lambda\mu_k}{1+\lambda\mu_k} \to \sigma_x^2 \quad \text{as } n \to \infty,$$

and therefore (a) is proved.

For (b) we write

$$n^{-1}\mathbf{x}^T(\mathbf{I} - \mathbf{S}_\lambda)^2\mathbf{x} = n^{-1}\sum_k (c_k^x + \xi_k^x)^2 \left(\frac{\lambda\mu_k}{1+\lambda\mu_k}\right)^2.$$

By Lemma 1.3 we have that

$$n^{-1}\sum_k (c_k^x)^2 \left(\frac{\lambda\mu_k}{1+\lambda\mu_k}\right)^2 = B_{\text{tp}}^2(f^x,\lambda) = \mathcal{O}(\lambda) = o(1).$$

For $a > 0$ we have $\frac{1}{1+a} \leq 1$ and $\frac{a}{1+a} \leq 1$ and therefore

$$1 - \left(\frac{a}{1+a}\right)^2 = \frac{(1+a)^2 - a^2}{(1+a)^2} = \frac{(1+a)+a}{(1+a)^2} \leq \frac{2}{1+a}.$$

Using this with $a = \lambda\mu_k$ we see from assumption (A3) and Lemma 1.2 that

$$\begin{aligned}
\sum_k (\xi_k^x)^2 - \sum_k (\xi_k^x)^2 \left(\frac{\lambda\mu_k}{1+\lambda\mu_k}\right)^2 &\leq \sup_k (\xi_k^x)^2 \sum_k \frac{2}{1+\lambda\mu_k} \\
&= \mathcal{O}\big((\log^2 n)n^{1/2-\tau}\big) = o(n).
\end{aligned}$$

So by assumption (A2), (b) is proved.

For (c) let $\mathbf{c} = \mathbf{\Phi}^T \mathbf{f}$ be the coefficients of $\mathbf{f}$ in the basis $\mathbf{\Phi}$. Then

$$n^{-1}\mathbf{x}^T(\mathbf{I}-\mathbf{S}_\lambda)\mathbf{f} = n^{-1}\sum_k (c_k^x c_k + \xi_k^x c_k)\frac{\lambda\mu_k}{1+\lambda\mu_k}.$$

For the term involving c_k^x, we use Cauchy-Schwarz and (2) to see that

$$\begin{aligned}\left|n^{-1}\sum_k c_k^x c_k \frac{\lambda\mu_k}{1+\lambda\mu_k}\right| &\leq \left(n^{-1}\sum_k (c_k^x)^2\right)^{1/2}\left(n^{-1}\sum_k\left(\frac{c_k\lambda\mu_k}{1+\lambda\mu_k}\right)^2\right)^{1/2} \\ &= \mathcal{O}\big((B^2_{\text{tp}}(f,\lambda))^{1/2}\big) = \mathcal{O}(\lambda^{1/2})\end{aligned}$$

by Lemma 1.3. For the term involving ξ_k^x, we use Cauchy-Schwarz again to obtain

$$\begin{aligned}\left|n^{-1}\sum_k \xi_k^x c_k \frac{\lambda\mu_k}{1+\lambda\mu_k}\right| &\leq \lambda^{1/2}\sup_k|\xi_k^x|\left|n^{-1}\sum_k c_k\mu_k^{1/2}\frac{(\lambda\mu_k)^{1/2}}{1+\lambda\mu_k}\right| \\ &\leq \lambda^{1/2}\sup_k|\xi_k^x|\left(n^{-1}\sum_k c_k^2\mu_k\right)^{1/2}\left(n^{-1}\sum_k\frac{\lambda\mu_k}{(1+\lambda\mu_k)^2}\right)^{1/2} \\ &\leq \mathcal{O}(\lambda^{1/2}\log n)\mathcal{O}(n^{-1/2}\lambda^{-d/4m}) = o(n^{-1/2})\end{aligned}$$

Here we have used assumption (A3), Lemma 1.2 (since $\frac{\lambda\mu_k}{(1+\lambda\mu_k)^2} \leq \frac{1}{1+\lambda\mu_k}$) and the fact that

$$n^{-1}\sum_k c_k^2\mu_k = \mathbf{f}^T\Gamma\mathbf{f} \leq |f|_m^2 < \infty$$

since $f \in H^m(\Omega)$. The rate of convergence of $o(n^{-1/2})$ follows from the fact that

$$n^{-1/2}(\log n)\lambda^{-d/4m+1/2} \approx n^{-1/2}(\log n)n^{-\delta(1-d/2m)/2} = o(n^{-1/2})$$

since $1 - d/2m > 0$. This proves (c).

For (d) we have that

$$n^{-1}\mathbf{x}^T\mathbf{S}_\lambda^2\mathbf{x} = n^1\sum_k (c_k^x + \xi_k^x)^2\frac{1}{(1+\lambda_x\mu_k)^2}.$$

For the term involving $(c_k^x)^2$ we see that

$$n^{-1}\sum_k (c_k^x)^2\frac{1}{(1+\lambda_x\mu_k)^2} \leq n^{-1}\sum_k (c_k^x)^2 = \mathcal{O}(1)$$

by (2). For the term involving $(\xi_k^x)^2$ we see from assumption (A3) and Lemma 1.2 that

$$\begin{aligned}n^{-1}\sum_k(\xi_k^x)^2\frac{1}{(1+\lambda_x\mu_k)^2} &\leq n^{-1}\sup_k(\xi_k^x)^2\sum_k\frac{1}{(1+\lambda_x\mu_k)^2} \\ &= \mathcal{O}\big((\log^2 n)n^{-1/2-\tau}\big) = \mathcal{O}(1).\end{aligned}$$

Hence $n^{-1}\mathbf{x}^T\mathbf{S}_\lambda^2\mathbf{x} = \mathcal{O}(1)$.

Proof of Lemma 1.5

As in the proof of Lemma 1.4 we write

$$n^{-1}\mathbf{x}^T(\mathbf{I}-\mathbf{S}_{\lambda_x})(\mathbf{I}-\mathbf{S}_{\lambda})(\mathbf{I}-\mathbf{S}_{\lambda_x})\mathbf{x} = n^{-1}\sum_k (c_k^x+\xi_k^x)^2\left(\frac{\lambda_x\mu_k}{1+\lambda_x\mu_k}\right)^2\frac{\lambda\mu_k}{1+\lambda\mu_k}$$

and once again, by Cauchy-Schwarz, we only need to consider the terms involving $(c_k^x)^2$ and $(\xi_k^x)^2$. Since $\frac{\lambda\mu_k}{1+\lambda\mu_k}\le 1$, Lemma 1.3 shows that

$$n^{-1}\sum_k (c_k^x)^2\left(\frac{\lambda_x\mu_k}{1+\lambda_x\mu_k}\right)^2\frac{\lambda\mu_k}{1+\lambda\mu_k}\le n^{-1}(\mathbf{f}^x)^T(\mathbf{I}-\mathbf{S}_{\lambda_x})^2\mathbf{f}^x = B_{\mathrm{tp}}^2(f^x,\lambda_x)=\mathcal{O}(\lambda_x)=o(1).$$

For the term involving $(\xi_k^x)^2$, firstly note that if $a_1,a_2,a_3>0$, then

$$\begin{aligned}1-\frac{a_1a_2a_3}{(1+a_1)(1+a_2)(1+a_3)} &= \frac{(1+a_1)(1+a_2)(1+a_3)-a_1a_2a_3}{(1+a_1)(1+a_2)(1+a_3)}\\ &= \frac{1+a_1+a_2+a_3+a_1a_2+a_1a_3+a_2a_3}{(1+a_1)(1+a_2)(1+a_3)}\\ &\le \frac{3}{1+a_1}+\frac{2}{1+a_2}+\frac{2}{1+a_3}\end{aligned}$$

where in the last step we have used the fact that $\frac{1}{1+a_i}\le 1$ and $\frac{a_i}{1+a_i}\le 1$ for all i. Using this with $a_1=a_2=\lambda_x\mu_k$ and $a_3=\lambda\mu_k$ we see that

$$\begin{aligned}\sum_k(\xi_k^x)^2-\sum_k(\xi_k^x)^2\left(\frac{\lambda_x\mu_k}{1+\lambda_x\mu_k}\right)^2\frac{\lambda\mu_k}{1+\lambda\mu_k} &\le \sup_k(\xi_k^x)^2\left(\sum_k\frac{5}{1+\lambda_x\mu_k}+\sum_k\frac{2}{1+\lambda_x\mu_k}\right)\\ &= \mathcal{O}(\log^2 n)\mathcal{O}(n^{1/2-\tau})=o(n)\end{aligned}$$

by assumption (A3) and Lemma 1.2. Therefore,

$$n^{-1}\sum_k(\xi_k^x)^2\left(\frac{\lambda_x\mu_k}{1+\lambda_x\mu_k}\right)^2\frac{\lambda\mu_k}{1+\lambda\mu_k}\to\sigma_x^2$$

by assumption (A2). This shows (a).

For (b) we have that

$$n^{-1}\mathbf{x}^T(\mathbf{I}-\mathbf{S}_{\lambda_x})(\mathbf{I}-\mathbf{S}_{\lambda})^2(\mathbf{I}-\mathbf{S}_{\lambda_x})\mathbf{x} = n^{-1}\sum_k (c_k^x+\xi_k^x)^2\left(\frac{\lambda_x\mu_k}{1+\lambda_x\mu_k}\right)^2\left(\frac{\lambda\mu_k}{1+\lambda\mu_k}\right)^2.$$

For the term involving $(c_k^x)^2$, the same argument as in (a) shows that this is $o(1)$. For the $(\xi_k^x)^2$ term we note that

$$1-\frac{a_1a_2a_3a_4}{(1+a_1)(1+a_2)(1+a_3)(1+a_4)}\le\frac{5}{1+a_1}+\frac{4}{1+a_2}+\frac{4}{1+a_3}+\frac{2}{1+a_4}$$

for $a_1,a_2,a_3,a_4>0$ and using this with $a_1=a_2=\lambda_x\mu_k$ and $a_3=a_4=\lambda\mu_k$ shows that

$$n^{-1}\sum_k(\xi_k^x)^2\left(\frac{\lambda_x\mu_k}{1+\lambda_x\mu_k}\right)^2\left(\frac{\lambda\mu_k}{1+\lambda\mu_k}\right)^2\to\sigma_x^2$$

as in (a). This proves (b).

For (c) let $\mathbf{c} = \mathbf{\Phi}^T \mathbf{f}$ be the coefficients of $\mathbf{f}$ in the basis $\mathbf{\Phi}$. Then

$$n^{-1}\mathbf{x}^T(\mathbf{I}-\mathbf{S}_{\lambda_x})(\mathbf{I}-\mathbf{S}_\lambda)\mathbf{f} = n^{-1}\sum_k (c_k^x c_k + \xi_k^x c_k)\frac{\lambda_x\mu_k}{1+\lambda_x\mu_k}\frac{\lambda\mu_k}{1+\lambda\mu_k}.$$

For the term involving c_k^x, we use Cauchy-Schwarz to see that

$$\begin{aligned}\left|n^{-1}\sum_k c_k^x c_k \frac{\lambda_x\mu_k}{1+\lambda_x\mu_k}\frac{\lambda\mu_k}{1+\lambda\mu_k}\right| &\le \left(n^{-1}\sum_k\left(\frac{c_k^x\lambda_x\mu_k}{1+\lambda_x\mu_k}\right)^2\right)^{1/2}\left(n^{-1}\sum_k\left(\frac{c_k\lambda\mu_k}{1+\lambda\mu_k}\right)^2\right)^{1/2} \\ &= \Big(B^2_{\text{tp}}(f^x,\lambda_x)B^2_{\text{tp}}(f,\lambda)\Big)^{1/2} = \mathcal{O}\big((\lambda_x\lambda)^{1/2}\big)\end{aligned}$$

by Lemma 1.3. For the term involving ξ_k^x, since $\frac{\lambda_x\mu_k}{1+\lambda_x\mu_k} \le 1$,

$$\left|n^{-1}\sum_k \xi_k^x c_k \frac{\lambda_x\mu_k}{1+\lambda_x\mu_k}\frac{\lambda\mu_k}{1+\lambda\mu_k}\right| \le \left|n^{-1}\sum_k \xi_k^x c_k\frac{\lambda\mu_k}{1+\lambda\mu_k}\right| = o(n^{-1/2})$$

by the proof of Lemma 1.4 (c). This proves (c).

For (d) we have that

$$n^{-1}\mathbf{x}^T(\mathbf{I}-\mathbf{S}_{\lambda_x})(\mathbf{I}-\mathbf{S}_\lambda)\mathbf{S}_{\lambda_x}\mathbf{x} = n^{-1}\sum_k (c_k^x+\xi_k^x)^2\frac{\lambda_x\mu_k}{(1+\lambda_x\mu_k)^2}\frac{\lambda\mu_k}{1+\lambda\mu_k}.$$

For the term involving $(c_k^x)^2$, Cauchy-Schwarz implies that

$$\begin{aligned}n^{-1}\sum_k (c_k^x)^2\frac{\lambda_x\mu_k}{(1+\lambda_x\mu_k)^2}\frac{\lambda\mu_k}{1+\lambda\mu_k} &\le n^{-1}\sum_k (c_k^x)^2\frac{\lambda_x\mu_k}{1+\lambda_x\mu_k}\frac{\lambda\mu_k}{1+\lambda\mu_k} \\ &\le \big(B^2_{\text{tp}}(f^x,\lambda_x)B^2_{\text{tp}}(f^x,\lambda)\big)^{1/2} = \mathcal{O}\big((\lambda\lambda_x)^{1/2}\big)\end{aligned}$$

by Lemma 1.3. For the term involving $(\xi_k^x)^2$ we use (A3) and Lemma 1.2 to see that

$$\begin{aligned}n^{-1}\sum_k (\xi_k^x)^2\frac{\lambda_x\mu_k}{(1+\lambda_x\mu_k)^2}\frac{\lambda\mu_k}{1+\lambda\mu_k} &\le \sup_k(\xi_k^x)^2 n^{-1}\sum_k\frac{1}{1+\lambda_x\mu_k} \\ &= \mathcal{O}\big((\log^2 n)n^{-1/2-\tau}\big) = o(n^{-1/2}).\end{aligned}$$

This proves (d)

For (e) we have that

$$n^{-1}\mathbf{x}^T\mathbf{S}_{\lambda_x}(\mathbf{I}-\mathbf{S}_\lambda)^2\mathbf{S}_{\lambda_x}\mathbf{x} = n^{-1}\sum_k (c_k^x+\xi_k^x)^2\frac{1}{(1+\lambda_x\mu_k)^2}\left(\frac{\lambda\mu_k}{1+\lambda\mu_k}\right)^2.$$

For the term involving $(c_k^x)^2$ we see that

$$\begin{aligned}n^{-1}\sum_k (c_k^x)^2\frac{1}{(1+\lambda_x\mu_k)^2}\left(\frac{\lambda\mu_k}{1+\lambda\mu_k}\right)^2 &\le n^{-1}\sum_k (c_k^x)^2\left(\frac{\lambda\mu_k}{1+\lambda\mu_k}\right)^2 \\ &= B^2_{\text{tp}}(f^x,\lambda) = \mathcal{O}(\lambda).\end{aligned}$$

For the term involving $(\xi_k^x)^2$

$$\begin{aligned}n^{-1}\sum_k (\xi_k^x)^2\frac{1}{(1+\lambda_x\mu_k)^2}\left(\frac{\lambda\mu_k}{1+\lambda\mu_k}\right)^2 &\le n^{-1}\sup_k(\xi_k^x)^2\sum_k\frac{1}{(1+\lambda_x\mu_k)^2} \\ &= \mathcal{O}\Big(n^{-1}(\log^2 n)\lambda_x^{-d/2m}\Big)\end{aligned}$$

by assumption (A3) and Lemma 1.2. This proves (e).

For (f) we write

$$n^{-1}\mathbf{x}^T\big[\mathbf{S}_\lambda + (\mathbf{I}-\mathbf{S}_\lambda)\mathbf{S}_{\lambda_x}\big]^T\big[\mathbf{S}_\lambda + (\mathbf{I}-\mathbf{S}_\lambda)\mathbf{S}_{\lambda_x}\big]\mathbf{x} \tag{3}$$
$$= n^{-1}\big(\mathbf{x}^T\mathbf{S}_\lambda^2\mathbf{x} + 2\mathbf{x}^T\mathbf{S}_\lambda(\mathbf{I}-\mathbf{S}_\lambda)\mathbf{S}_{\lambda_x}\mathbf{x} + \mathbf{x}^T\mathbf{S}_{\lambda_x}(\mathbf{I}-\mathbf{S}_\lambda)^2\mathbf{S}_{\lambda_x}\big).$$

For the first term in (3), $n^{-1}\mathbf{x}^T\mathbf{S}_\lambda^2\mathbf{x} = \mathcal{O}(1)$ by Lemma 1.4 (d). For the second term in (3) we see that

$$\begin{aligned} n^{-1}\mathbf{x}^T\mathbf{S}_\lambda(\mathbf{I}-\mathbf{S}_\lambda)\mathbf{S}_{\lambda_x}\mathbf{x} &= n^{-1}\sum_k (c_k^x+\xi_k^x)^2\frac{1}{1+\lambda_x\mu_k}\frac{\lambda\mu_k}{(1+\lambda\mu_k)^2} \\ &\leq n^{-1}\sum_k (c_k^x+\xi_k^x)^2\frac{\lambda\mu_k}{1+\lambda\mu_k} = n^{-1}\mathbf{x}^T(\mathbf{I}-\mathbf{S}_\lambda)\mathbf{x} = \mathcal{O}(1) \end{aligned}$$

by Lemma 1.4 (a). From (e), the third term in (3) is given by

$$\begin{aligned} n^{-1}\mathbf{x}^T\mathbf{S}_{\lambda_x}(\mathbf{I}-\mathbf{S}_\lambda)^2\mathbf{S}_{\lambda_x} &= \mathcal{O}(\lambda) + \mathcal{O}(n^{-1}\lambda_x^{-d/2m}\log^2 n) \\ &\approx \mathcal{O}(n^{-\delta}) + \mathcal{O}(n^{-(1-\delta_x d/2m)}\log^2 n) = \mathcal{O}(1). \end{aligned}$$

This proves (f).

3 Web Appendix C: Main asymptotic results

This appendix details the main results of our asymptotic analysis referred to in Section 3 of the paper.

3.1 Asymptotic results for the spatial model

In the model (1) of the paper, spatial correlation is modeled through smoothing of the term f. Without the smoothing penalty, the model is an ordinary linear model in which all effect estimates are unbiased. Therefore, bias in the covariate effect estimate arises as a direct result of smoothing. Rice (1986) showed for dimension $d = 1$ that, while this bias is asymptotically 0 as $n \to \infty$, the rate of convergence may be slow. More specifically, we cannot ensure that the bias converges faster than the standard deviation if the smoothing parameter λ converges at the optimal rate (minimizing the AMSE of the estimated spatial effect). Therefore, the bias can in practice become disproportionately large. Here, we generalize Rice's results and see that the problem of potentially excessive bias in $\widehat{\beta}$ persists in models where the spatial domain has dimension $d \geq 1$. As an aside, we note that, as in the $d = 1$ case, the rate of convergence of the variance of $\widehat{\beta}$, is the same as that in a model with no smoothing penalty.

Theorem 3.1. *Suppose $\lambda \approx n^{-\delta}$ for some $0 < \delta < 1$, $f, f^x \in H^m(\Omega)$ are bounded and $m \geq d$. Then for the partial thin plate spline estimate of β we have that*

(a) $\mathrm{E}(\widehat{\beta}) - \beta = o(n^{-1/2}) + \mathcal{O}(\lambda^{1/2})$,

(b) $n\mathrm{Var}(\widehat{\beta}) \to \sigma^2/\sigma_x^2$ *as* $n \to \infty$.

In particular, $\mathrm{Var}(\widehat{\beta}) = \mathcal{O}(n^{-1})$ *and we need* $\lambda = o(n^{-1})$ *to ensure that the bias converges faster than the standard deviation of* $\widehat{\beta}$.

Proof. Let $\mathbf{f} = (f(\mathbf{t}_1), \ldots, f(\mathbf{t}_n))^T$. Since $\mathrm{E}(\mathbf{y}) = \beta\mathbf{x} + \mathbf{f}$, the expression (3) in the paper shows that

$$\begin{aligned} \mathrm{E}(\widehat{\beta}) - \beta &= \big(\mathbf{x}^T(\mathbf{I}-\mathbf{S}_\lambda)\mathbf{x}\big)^{-1}\mathbf{x}^T(\mathbf{I}-\mathbf{S}_\lambda)(\beta\mathbf{x}+\mathbf{f}) - \beta \\ &= \big(n^{-1}\mathbf{x}^T(\mathbf{I}-\mathbf{S}_\lambda)\mathbf{x}\big)^{-1}\big(n^{-1}\mathbf{x}^T(\mathbf{I}-\mathbf{S}_\lambda)\mathbf{f}\big) \\ &= o(n^{-1/2}) + \mathcal{O}(\lambda^{1/2}) \end{aligned}$$

by Lemma 1.4 (a) and (c).

Similarly, since $\mathrm{Var}(\mathbf{y}) = \sigma^2\mathbf{I}$, (3) in the paper shows that

$$\begin{aligned} n\mathrm{Var}(\widehat{\beta}) &= n\sigma^2\big(\mathbf{x}^T(\mathbf{I}-\mathbf{S}_\lambda)\mathbf{x}\big)^{-1}\mathbf{x}^T(\mathbf{I}-\mathbf{S}_\lambda)^2\mathbf{x}\big(\mathbf{x}^T(\mathbf{I}-\mathbf{S}_\lambda)\mathbf{x}\big)^{-1} \\ &= \sigma^2\big(n^{-1}\mathbf{x}^T(\mathbf{I}-\mathbf{S}_\lambda)\mathbf{x}\big)^{-1}\big(n^{-1}\mathbf{x}^T(\mathbf{I}-\mathbf{S}_\lambda)^2\mathbf{x}\big)\big(n^{-1}\mathbf{x}^T(\mathbf{I}-\mathbf{S}_\lambda)\mathbf{x}\big)^{-1} \\ &\to \sigma^2/\sigma_x^2 \quad \text{as } n\to\infty \end{aligned}$$

by Lemma 1.4 (a) and (b). □

Theorem 3.2. *Suppose $\lambda \approx n^{-\delta}$ for some $0 < \delta < 1$, $f, f^x \in H^m(\Omega)$ are bounded and $m \geq d$. Then the average squared bias $B^2(f,\lambda)$ and average variance $V(f,\lambda)$ of the partial thin plate spline estimate of f satisfy*

(a) $B^2(f,\lambda) = n^{-1}\sum_i(\mathrm{E}(\widehat{f}_i) - f(\mathbf{t}_i))^2 = \mathcal{O}(\lambda)$,

(b) $V(f,\lambda) = n^{-1}\sum_i \mathrm{Var}(\widehat{f}_i) = \mathcal{O}(n^{-1}\lambda^{-d/2m})$.

In particular, the optimal rate for λ in terms of minimizing $\mathrm{AMSE}(\widehat{\mathbf{f}})$ is $\lambda = \mathcal{O}(n^{-2m/(2m+d)})$, and when λ converges at this optimal rate, $\mathrm{AMSE}(\widehat{\mathbf{f}}) = \mathcal{O}(n^{-2m/(2m+d)})$.

Proof. Let $\mathbf{f} = (f(\mathbf{t}_1),\ldots,f(\mathbf{t}_n))^T$ and $\boldsymbol{\epsilon} = (\epsilon_1,\ldots,\epsilon_n)^T$ so that $\mathbf{y} = \beta\mathbf{x} + \mathbf{f} + \boldsymbol{\epsilon}$.

Since $\widehat{\mathbf{f}} = \mathbf{S}_\lambda(\mathbf{y} - \widehat{\beta}\mathbf{x})$ by (3) in the paper,

$$\mathrm{E}(\widehat{\mathbf{f}}) - \mathbf{f} = -\big(\mathrm{E}(\widehat{\beta}) - \beta\big)\mathbf{S}_\lambda\mathbf{x} - (\mathbf{I}-\mathbf{S}_\lambda)\mathbf{f}.$$

We therefore see that

$$\begin{aligned} B^2(f,\lambda) &= n^{-1}\|\mathrm{E}(\widehat{\mathbf{f}}) - \mathbf{f}\|^2 \\ &\leq n^{-1}\big\|\big(\mathrm{E}(\widehat{\beta})-\beta\big)\mathbf{S}_\lambda\mathbf{x}\big\|^2 + n^{-1}\|(\mathbf{I}-\mathbf{S}_\lambda)\mathbf{f}\|^2 \\ &= \big(\mathrm{E}(\widehat{\beta})-\beta\big)^2 n^{-1}\mathbf{x}^T\mathbf{S}_\lambda^2\mathbf{x} + B^2_{\mathrm{tp}}(f,\lambda) \\ &= \big(o(n^{-1}) + \mathcal{O}(\lambda)\big)\mathcal{O}(1) + \mathcal{O}(\lambda) = \mathcal{O}(\lambda) \end{aligned}$$

by Theorem 1(a), Lemma 1.4 (d) and Lemma 1.3. This proves part (a).

For (b), firstly note that

$$\widehat{\mathbf{f}} - \mathrm{E}(\widehat{\mathbf{f}}) = \mathbf{S}_\lambda\boldsymbol{\epsilon} - \big(\widehat{\beta} - \mathrm{E}(\widehat{\beta})\big)\mathbf{S}_\lambda\mathbf{x}.$$

We therefore see that

$$\begin{aligned} V(f,\lambda) &= n^{-1}\mathrm{E}\big(\|\widehat{\mathbf{f}} - \mathrm{E}(\widehat{\mathbf{f}})\|^2\big) \\ &\leq n^{-1}\mathrm{E}(\boldsymbol{\epsilon}^T\mathbf{S}_\lambda^2\boldsymbol{\epsilon}) + \mathrm{E}\big[(\widehat{\beta} - \mathrm{E}(\widehat{\beta}))^2\big]n^{-1}\mathbf{x}^T\mathbf{S}_\lambda^2\mathbf{x} \\ &= n^{-1}\sigma^2\mathrm{Tr}(\mathbf{S}_\lambda^2) + \mathrm{Var}(\widehat{\beta})\mathcal{O}(1) \\ &= \mathcal{O}(n^{-1}\lambda^{-d/2m}) + \mathcal{O}(n^{-1}) = \mathcal{O}(n^{-1}\lambda^{-d/2m}) \end{aligned}$$

by Lemma 1.2, Theorem 1(b) and Lemma 1.4 (d). This proves part (b).

Finally, recall that

$$\mathrm{AMSE}(\widehat{\mathbf{f}}) = B^2(f,\lambda) + V(f,\lambda).$$

From the above, we see that the bias term increases with λ while the variance term decreases with λ so that the optimal rate for minimizing $\mathrm{AMSE}(\widehat{\mathbf{f}})$ is achieved when $\mathcal{O}(\lambda) = \mathcal{O}(n^{-1}\lambda^{-d/2m})$. This leads to an optimal rate of $\lambda = \mathcal{O}(n^{-2m/(2m+d)})$. At this rate for λ, $B^2(f,\lambda)$ and $V(f,\lambda)$ converge at the same rate of $\mathcal{O}(n^{-2m/(2m+d)})$. □

We have therefore proved the following result which shows that we cannot avoid the potential for excessive bias in $\widehat{\beta}$, unless λ converges at a rate slower than the optimal rate of convergence, i.e. unless the smooth term is undersmoothed.

Corollary 3.3. *Suppose $\lambda \approx n^{-\delta}$ for some $0 < \delta < 1$, $f, f^x \in H^m(\Omega)$ are bounded and $m \geq d$. The optimal rate of convergence for λ in terms of minimizing* $\mathrm{AMSE}(\widehat{\mathbf{f}})$ *is slower than the required rate of $o(n^{-1})$ for ensuring that the bias of $\widehat{\beta}$ converges faster than the standard deviation of the estimate.*

3.2 Asymptotic results for the spatial+ model

In dimension $d = 1$, Chen and Shiau (1991) show that for the model (5) of the paper, the problems identified by Rice disappear. That is, when the parameters λ and λ_x converge at the optimal rate (for minimizing the AMSE of the estimated spatial effect), the bias of the covariate effect estimate $\widehat{\beta}^+$ converges to 0 faster than the standard deviation and, therefore, does not become disproportionately large. We now generalize these results to dimensions $d \geq 1$.

Theorem 3.4. *Suppose $\lambda \approx n^{-\delta}, \lambda_x \approx n^{-\delta_x}$ for some $0 < \delta, \delta_x < 1$, $f, f^x \in H^m(\Omega)$ are bounded and $m \geq d$. Then for the spatial+ estimate of β we have that*

(a) $\mathrm{E}(\widehat{\beta}^+) - \beta = o(n^{-1/2}) + \mathcal{O}((\lambda\lambda_x)^{1/2})$,

(b) $n\mathrm{Var}(\widehat{\beta}^+) \to \sigma^2/\sigma_x^2$ *as* $n \to \infty$.

In particular, $\mathrm{Var}(\widehat{\beta}^+) = \mathcal{O}(n^{-1})$ *and we need $\lambda\lambda_x = o(n^{-1})$ to ensure that the bias converges faster than the standard deviation of $\widehat{\beta}^+$.*

Proof. Let

$$\begin{aligned}
\mathbf{b} &= (\mathbf{I} - \mathbf{S}_\lambda)(\mathbf{I} - \mathbf{S}_{\lambda_x})\mathbf{x} \\
a_1 &= n^{-1}\mathbf{b}^T(\mathbf{I} - \mathbf{S}_{\lambda_x})\mathbf{x} = n^{-1}\mathbf{x}^T(\mathbf{I} - \mathbf{S}_{\lambda_x})(\mathbf{I} - \mathbf{S}_\lambda)(\mathbf{I} - \mathbf{S}_{\lambda_x})\mathbf{x} \\
a_2 &= n^{-1}\mathbf{b}^T\mathbf{b} = n^{-1}\mathbf{x}^T(\mathbf{I} - \mathbf{S}_{\lambda_x})(\mathbf{I} - \mathbf{S}_\lambda)^2(\mathbf{I} - \mathbf{S}_{\lambda_x})\mathbf{x}.
\end{aligned}$$

By Lemma 1.5 (a) and (b), $a_1 \to \sigma_x^2$ and $a_2 \to \sigma_x^2$ as $n \to \infty$. From (7) in the paper we see that

$$\widehat{\beta}^+ = (na_1)^{-1}\mathbf{b}^T\mathbf{y}.$$

Therefore, since $\mathrm{E}(\mathbf{y}) = \beta\mathbf{x} + \mathbf{f}$ where $\mathbf{f} = (f(\mathbf{t}_1), \ldots, f(\mathbf{t}_n))$,

$$\begin{aligned}
\mathrm{E}(\widehat{\beta}^+) - \beta &= (na_1)^{-1}\big((\mathbf{b}^T\mathbf{x} - na_1)\beta + \mathbf{b}^T\mathbf{f}\big) \\
&= (na_1)^{-1}\big(\mathbf{b}^T\mathbf{S}_{\lambda_x}\mathbf{x}\beta + \mathbf{b}^T\mathbf{f}\big) \\
&= a_1^{-1}\big(n^{-1}\mathbf{x}^T(\mathbf{I} - \mathbf{S}_{\lambda_x})(\mathbf{I} - \mathbf{S}_\lambda)\mathbf{S}_{\lambda_x}\mathbf{x}\beta + n^{-1}\mathbf{x}^T(\mathbf{I} - \mathbf{S}_{\lambda_x})(\mathbf{I} - \mathbf{S}_\lambda)\mathbf{f}\big) \\
&= o(n^{-1/2}) + \mathcal{O}((\lambda\lambda_x)^{1/2})
\end{aligned}$$

by Lemma 1.5 (d) and (c). This proves part (a).

For part (b), since $\mathrm{Var}(\mathbf{y}) = \sigma^2\mathbf{I}$, we see that

$$\begin{aligned}
n\mathrm{Var}(\widehat{\beta}^+) &= n(na_1)^{-2}\mathbf{b}^T(\sigma^2\mathbf{I})\mathbf{b} \\
&= (\sigma^2 a_2)/a_1^2 \\
&\to \sigma^2/\sigma_x^2 \quad \text{as } n \to \infty.
\end{aligned}$$

This proves (b). □

Theorem 3.5. *Suppose $\lambda \approx n^{-\delta}, \lambda_x \approx n^{-\delta_x}$ for some $0 < \delta, \delta_x < 1$, $f, f^x \in H^m(\Omega)$ are bounded and $m \geq d$. Then the average squared bias $B_+^2(f, \lambda, \lambda_x)$ and average variance $V_+(f, \lambda, \lambda_x)$ of the spatial+ estimate of f satisfy*

(a) $B_+^2(f, \lambda, \lambda_x) = n^{-1} \sum_i (\mathrm{E}(\widehat{f}_i^+) - f(\mathbf{t}_i))^2 = \mathcal{O}(\lambda) + \mathcal{O}(n^{-1}\lambda_x^{-d/2m} \log^2 n)$,

(b) $V_+(f, \lambda, \lambda_x) = n^{-1} \sum_i \mathrm{Var}(\widehat{f}_i^+) = \mathcal{O}(n^{-1}\lambda^{-d/2m})$.

In particular, the optimal rates for λ and λ_x in terms of minimizing $\mathrm{AMSE}(\widehat{\mathbf{f}}^+)$ are given by $\lambda = \mathcal{O}(n^{-2m/(2m+d)})$ and $\lambda_x = \mathcal{O}(n^{-2m/(2m+d)}(\log n)^{4m/d})$, assuming the convergence rates for $B_+^2(f, \lambda, \lambda)$ and $V_+(f, \lambda, \lambda_x)$ are equal. When λ and λ_x converge at these rates, $\mathrm{AMSE}(\widehat{\mathbf{f}}^+) = \mathcal{O}(n^{-2m/(2m+d)})$.

Proof. Let $\mathbf{f} = (f(\mathbf{t}_1), \ldots, f(\mathbf{t}_n))^T$ and $\boldsymbol{\epsilon} = (\epsilon_1, \ldots, \epsilon_n)^T$ so that $\mathbf{y} = \beta\mathbf{x} + \mathbf{f} + \boldsymbol{\epsilon}$. Since by (8) in the paper

$$\widehat{\mathbf{f}}^+ = \mathbf{S}_\lambda \mathbf{y} - \big(\mathbf{S}_\lambda + (\mathbf{I} - \mathbf{S}_\lambda)\mathbf{S}_{\lambda_x}\big)\widehat{\beta}^+\mathbf{x},$$

we have that

$$\mathrm{E}(\widehat{\mathbf{f}}^+) - \mathbf{f} = -(\mathbf{I} - \mathbf{S}_\lambda)\mathbf{f} - (\mathrm{E}(\widehat{\beta}^+) - \beta)\big(\mathbf{S}_\lambda + (\mathbf{I} - \mathbf{S}_\lambda)\mathbf{S}_{\lambda_x}\big)\mathbf{x} - \beta(\mathbf{I} - \mathbf{S}_\lambda)\mathbf{S}_{\lambda_x}\mathbf{x}.$$

Since $n^{-1}\big\|(\mathbf{I} - \mathbf{S}_\lambda)\mathbf{f}\big\|^2 = B_{\mathrm{tp}}^2(f, \lambda)$, we therefore see that

$$\begin{aligned}
B_+^2(f, \lambda, \lambda_x) &= n^{-1}\|\mathrm{E}(\widehat{\mathbf{f}}^+) - \mathbf{f}\|^2 \\
&\leq n^{-1}\big\|(\mathbf{I} - \mathbf{S}_\lambda)\mathbf{f}\big\|^2 + \big(\mathrm{E}(\widehat{\beta}^+) - \beta\big)^2 n^{-1}\big\|\big(\mathbf{S}_\lambda + (\mathbf{I} - \mathbf{S}_\lambda)\mathbf{S}_{\lambda_x}\big)\mathbf{x}\big\|^2 \\
&\quad + \beta^2 n^{-1}\big\|(\mathbf{I} - \mathbf{S}_\lambda)\mathbf{S}_{\lambda_x}\mathbf{x}\big\|^2 \\
&= \mathcal{O}(\lambda) + \big(o(n^{-1}) + \mathcal{O}(\lambda\lambda_x)\big)\mathcal{O}(1) + \mathcal{O}(\lambda) + \mathcal{O}(n^{-1}\lambda_x^{d/2m} \log^2 n) \\
&= \mathcal{O}(\lambda) + \mathcal{O}(n^{-1}\lambda_x^{-d/2m} \log^2 n)
\end{aligned}$$

by Lemma 1.3, Theorem 3(a) and Lemma 1.4 (f) and (e). This proves part (a).

For (b), note that

$$\widehat{\mathbf{f}}^+ - \mathrm{E}(\widehat{\mathbf{f}}^+) = \mathbf{S}_\lambda\boldsymbol{\epsilon} - \big(\widehat{\beta}^+ - \mathrm{E}(\widehat{\beta}^+)\big)\big(\mathbf{S}_\lambda + (\mathbf{I} - \mathbf{S}_\lambda)\mathbf{S}_{\lambda_x}\big)\mathbf{x}.$$

We therefore see that

$$\begin{aligned}
V_+(f, \lambda, \lambda_x) &= n^{-1}\mathrm{E}\big[\|\widehat{\mathbf{f}}^+ - \mathrm{E}(\widehat{\mathbf{f}}^+)\|^2\big] \\
&\leq n^{-1}\mathrm{E}[\boldsymbol{\epsilon}^T\mathbf{S}_\lambda^2\boldsymbol{\epsilon}] - \mathrm{E}\big[(\widehat{\beta}^+ - \mathrm{E}(\widehat{\beta}^+))^2\big] n^{-1}\big\|\big(\mathbf{S}_\lambda + (\mathbf{I} - \mathbf{S}_\lambda)\mathbf{S}_{\lambda_x}\big)\mathbf{x}\big\|^2 \\
&= n^{-1}\sigma^2\mathrm{Tr}(\mathbf{S}_\lambda^2) + \mathrm{Var}(\widehat{\beta}^+)\mathcal{O}(1) \\
&= \mathcal{O}(n^{-1}\lambda^{-d/2m}) + \mathcal{O}(n^{-1}) = \mathcal{O}(n^{-1}\lambda^{-d/2m})
\end{aligned}$$

by Lemma 1.5 (f), Lemma 1.2 and Theorem 3(b). This proves part (b).

Finally, recall that

$$\mathrm{AMSE}(\widehat{\mathbf{f}}^+) = B_+^2(f, \lambda, \lambda_x) + V_+(f, \lambda, \lambda_x).$$

From the above we see that the bias term increases with λ while the variance term decreases with λ so that the optimal rate for minimizing $\mathrm{AMSE}(\widehat{\mathbf{f}}^+)$ is achieved when $\mathcal{O}(\lambda) = \mathcal{O}(n^{-1}\lambda^{-d/2m})$. This leads to an optimal rate of $\lambda = \mathcal{O}(n^{-2m/(2m+d)})$. Since we have assumed that the convergence rates for $B_+^2(f, \lambda, \lambda_x)$ and $V_+(f, \lambda, \lambda_x)$ are equal, the optimal rate for λ_x is then obtained when $\mathcal{O}(n^{-1}\lambda_x^{-d/2m} \log^2 n) = \mathcal{O}(n^{-2m/(2m+d)})$ which leads to $\mathcal{O}(\lambda_x) = n^{-2m/(2m+d)}(\log n)^{4m/d}$. □

From this we obtain the following result which shows that, unlike $\widehat{\beta}$, the estimate $\widehat{\beta}^+$ does not need undersmoothing to avoid excessive bias.

Corollary 3.6. *Suppose $\lambda \approx n^{-\delta}, \lambda_x \approx n^{-\delta_x}$ for some $0 < \delta, \delta_x < 1$, $f, f^x \in H^m(\Omega)$ are bounded and $m \geq d$. If λ and λ_x converge at the optimal rates in terms of minimizing $\mathrm{AMSE}(\widehat{\mathbf{f}}^+)$, then $\lambda\lambda_x = o(n^{-1})$. In particular, the optimal rates for λ and λ_x ensure that the bias of the spatial+ estimate $\widehat{\beta}^+$ converges faster than the standard deviation of the estimate.*

Proof. Theorem 3(b) shows that we need $\mathrm{E}(\widehat{\beta}^+) - \beta = o(n^{-1/2})$ to ensure that the bias converges faster than the standard deviation. Part (a) of the same theorem shows that this required rate can be achieved if $\lambda\lambda_x = o(n^{-1})$. Suppose λ and λ_x converge at their optimal rates from Theorem 4. Then since for any $\epsilon > 0$,

$$n^{-2m/(2m+d)}(\log n)^{4m/d} = o(n^{-2m/(2m+d)+\epsilon}),$$

we have that

$$\lambda\lambda_x = o(n^{-4m/(2m+d)+\epsilon}) = o(n^{-1})$$

if we choose $\epsilon = \frac{2m-d}{2m+d}$. This proves the result. □

4 Web Appendix D: Partial residual estimates

In this appendix we consider, as an aside, the asymptotic behaviour of the partial residual estimates introduced by Denby (1986) and, independently, by Speckman (1988), which are the estimates we obtain using the gSEM approach of Thaden and Kneib (2018). Here we adapt the method used in Sections 3.2 and 3.3 of the paper for estimates in the spatial and spatial+ models to show how the asymptotic results in Chen and Shiau (1991) for the partial residual estimates generalize from the one-dimensional model to dimensions $d \geq 1$. We show that, as is the case for the spatial+ model, the smoothing-induced bias in the covariate effect estimate goes to 0 faster than the standard deviation, i.e. the partial residual estimates also avoid the problem of disproportionate smoothing-induced bias.

For a given value $\lambda > 0$ of the smoothing parameter, the partial residual estimates for the covariate effect β and the unknown smooth effect $\mathbf{f} = (f(\mathbf{t}_1), \ldots, f(\mathbf{t}_n))^T$ in the model (1) of the paper, are defined as

$$\begin{aligned} \widehat{\beta}_{\mathrm{pr}} &= \left(\mathbf{x}^T(\mathbf{I} - \mathbf{S}_\lambda)^2\mathbf{x}\right)^{-1}\mathbf{x}^T(\mathbf{I} - \mathbf{S}_\lambda)^2\mathbf{y}, \\ \widehat{\mathbf{f}}_{\mathrm{pr}} &= \mathbf{S}_\lambda(\mathbf{y} - \widehat{\beta}_{\mathrm{pr}}\mathbf{x}) \end{aligned} \tag{4}$$

where $\mathbf{S}_\lambda$ is the smoother matrix. A similar argument to that of Section 2.2 of the paper shows that these estimates are the ones we would obtain in the gSEM if, for simplicity, we used the same smoothing parameter in all regressions. That is, the estimate $\widehat{\beta}_{\mathrm{pr}}$ is the same as the estimated effect in the linear model given by

$$r_i^y = \beta r_i^x + \epsilon_i, \quad \epsilon_i \underset{\mathrm{iid}}{\sim} N(0, \sigma^2)$$

where $\mathbf{r}^x = (\mathbf{I} - \mathbf{S}_\lambda)\mathbf{x}$ and $\mathbf{r}^y = (\mathbf{I} - \mathbf{S}_\lambda)\mathbf{y}$ are the residuals after fitting a thin plate spline to $\mathbf{x}$ and $\mathbf{y}$, respectively.

Minor adjustments to the proofs of Theorems 1 and 2 and Corollary 1 for the spatial model estimates lead to the following results. These results show that the asymptotic behaviour of the estimates $\widehat{\beta}_{\mathrm{pr}}$ and $\widehat{\mathbf{f}}_{\mathrm{pr}}$ is the same as that of the corresponding spatial model estimates, except for the rate of convergence of the bias of the covariate effect estimate $\widehat{\beta}_{\mathrm{pr}}$. More specifically, $\mathrm{E}(\widehat{\beta}_{\mathrm{pr}}) - \beta = o(n^{-1/2}) + \mathcal{O}(\lambda)$, whereas $\mathrm{E}(\widehat{\beta}) - \beta = o(n^{-1/2}) + \mathcal{O}(\lambda^{1/2})$ and this difference is enough to ensure that the bias converges faster than the standard deviation when λ converges at the optimal rate (for minimizing the AMSE of the estimated spatial effect).

Theorem 4.1. *Suppose $\lambda \approx n^{-\delta}$ for some $0 < \delta < 1$, $f, f^x \in H^m(\Omega)$ are bounded and $m \geq d$. Then for the partial residual estimate of β we have that*

(a) $\mathrm{E}(\widehat{\beta}_{\mathrm{pr}}) - \beta = o(n^{-1/2}) + \mathcal{O}(\lambda)$,

(b) $n\text{Var}(\widehat{\beta}_{\text{pr}}) \to \sigma^2/\sigma_x^2$ *as* $n \to \infty$.

In particular, $\text{Var}(\widehat{\beta}_{\text{pr}}) = \mathcal{O}(n^{-1})$ *and we need* $\lambda = o(n^{-1/2})$ *to ensure that the bias converges faster than the standard deviation of* $\widehat{\beta}_{\text{pr}}$.

Proof. Let $\mathbf{f} = (f(\mathbf{t}_1), \ldots, f(\mathbf{t}_n))^T$. Since $\text{E}(\mathbf{y}) = \beta\mathbf{x} + \mathbf{f}$, the expression (4) shows that

$$\begin{aligned} \text{E}(\widehat{\beta}_{\text{pr}}) - \beta &= \big(\mathbf{x}^T(\mathbf{I} - \mathbf{S}_\lambda)^2\mathbf{x}\big)^{-1}\mathbf{x}^T(\mathbf{I} - \mathbf{S}_\lambda)^2(\beta\mathbf{x} + \mathbf{f}) - \beta \\ &= \big(n^{-1}\mathbf{x}^T(\mathbf{I} - \mathbf{S}_\lambda)^2\mathbf{x}\big)^{-1}\big(n^{-1}\mathbf{x}^T(\mathbf{I} - \mathbf{S}_\lambda)^2\mathbf{f}\big) \\ &= o(n^{-1/2}) + \mathcal{O}(\lambda) \end{aligned}$$

by Lemma 1.4 (b) and Lemma 1.5 (c).

Similarly, since $\text{Var}(\mathbf{y}) = \sigma^2\mathbf{I}$, (4) shows that

$$\begin{aligned} n\text{Var}(\widehat{\beta}_{\text{pr}}) &= n\sigma^2\big(\mathbf{x}^T(\mathbf{I} - \mathbf{S}_\lambda)^2\mathbf{x}\big)^{-1}\mathbf{x}^T(\mathbf{I} - \mathbf{S}_\lambda)^4\mathbf{x}\big(\mathbf{x}^T(\mathbf{I} - \mathbf{S}_\lambda)^2\mathbf{x}\big)^{-1} \\ &= \sigma^2\big(n^{-1}\mathbf{x}^T(\mathbf{I} - \mathbf{S}_\lambda)^2\mathbf{x}\big)^{-1}\big(n^{-1}\mathbf{x}^T(\mathbf{I} - \mathbf{S}_\lambda)^4\mathbf{x}\big)\big(n^{-1}\mathbf{x}^T(\mathbf{I} - \mathbf{S}_\lambda)^2\mathbf{x}\big)^{-1} \\ &\to \sigma^2/\sigma_x^2 \quad \text{as } n \to \infty \end{aligned}$$

by Lemma 1.4 (b) and Lemma 1.5 (b). □

Theorem 4.2. *Suppose* $\lambda \approx n^{-\delta}$ *for some* $0 < \delta < 1$, $f, f^x \in H^m(\Omega)$ *are bounded and* $m \geq d$. *Then the average squared bias* $B_{\text{pr}}^2(f, \lambda)$ *and average variance* $V_{\text{pr}}(f, \lambda)$ *of the partial residual estimate of* f *satisfy*

(a) $B_{\text{pr}}^2(f, \lambda) = n^{-1}\sum_i(\text{E}((\widehat{\mathbf{f}}_{\text{pr}})_i) - f(\mathbf{t}_i))^2 = \mathcal{O}(\lambda)$,

(b) $V_{\text{pr}}(f, \lambda) = n^{-1}\sum_i \text{Var}((\widehat{\mathbf{f}}_{\text{pr}})_i) = \mathcal{O}(n^{-1}\lambda^{-d/2m})$.

In particular, the optimal rate for λ *in terms of minimizing* $\text{AMSE}(\widehat{\mathbf{f}}_{\text{pr}})$ *is* $\lambda = \mathcal{O}(n^{-2m/(2m+d)})$, *and when* λ *converges at this optimal rate,* $\text{AMSE}(\widehat{\mathbf{f}}_{\text{pr}}) = \mathcal{O}(n^{-2m/(2m+d)})$.

Proof. Let $\mathbf{f} = (f(\mathbf{t}_1), \ldots, f(\mathbf{t}_n))^T$ and $\boldsymbol{\epsilon} = (\epsilon_1, \ldots, \epsilon_n)^T$ so that $\mathbf{y} = \beta\mathbf{x} + \mathbf{f} + \boldsymbol{\epsilon}$.

By (4), $\widehat{\mathbf{f}}_{\text{pr}} = \mathbf{S}_\lambda(\mathbf{y} - \widehat{\beta}_{\text{pr}}\mathbf{x})$ has the same format as the corresponding partial thin plate spline estimate, and therefore,

$$\text{E}(\widehat{\mathbf{f}}_{\text{pr}}) - \mathbf{f} = -\big(\text{E}(\widehat{\beta}_{\text{pr}}) - \beta\big)\mathbf{S}_\lambda\mathbf{x} - (\mathbf{I} - \mathbf{S}_\lambda)\mathbf{f}$$

and

$$\widehat{\mathbf{f}}_{\text{pr}} - \text{E}(\widehat{\mathbf{f}}_{\text{pr}}) = \mathbf{S}_\lambda\boldsymbol{\epsilon} - \big(\widehat{\beta}_{\text{pr}} - \text{E}(\widehat{\beta}_{\text{pr}})\big)\mathbf{S}_\lambda\mathbf{x}.$$

as in the proof of Theorem 2. For the derivation of $B_{\text{pr}}^2(f, \lambda)$ and $V_{\text{pr}}(f, \lambda)$, we can therefore apply the same proof where the only adjustment needed is the rate of convergence of the bias $\text{E}(\widehat{\beta}_{\text{pr}}) - \beta$.

$$\begin{aligned} B_{\text{pr}}^2(f, \lambda) &= n^{-1}\|\text{E}(\widehat{\mathbf{f}}_{\text{pr}}) - \mathbf{f}\|^2 \\ &\leq n^{-1}\big\|\big(\text{E}(\widehat{\beta}_{\text{pr}}) - \beta\big)\mathbf{S}_\lambda\mathbf{x}\big\|^2 + n^{-1}\|(\mathbf{I} - \mathbf{S}_\lambda)\mathbf{f}\|^2 \\ &= \big(\text{E}(\widehat{\beta}_{\text{pr}}) - \beta\big)^2 n^{-1}\mathbf{x}^T\mathbf{S}_\lambda^2\mathbf{x} + B_{\text{tp}}^2(f, \lambda) \\ &= \big(o(n^{-1}) + \mathcal{O}(\lambda^2)\big)\mathcal{O}(1) + \mathcal{O}(\lambda) = \mathcal{O}(\lambda) \end{aligned}$$

by Theorem 4.1 (a), Lemma 1.4 (d) and Lemma 1.3. This proves part (a).

$$\begin{aligned} V_{\text{pr}}(f, \lambda) &= n^{-1}\text{E}\big(\|\widehat{\mathbf{f}}_{\text{pr}} - \text{E}(\widehat{\mathbf{f}}_{\text{pr}})\|^2\big) \\ &\leq n^{-1}\text{E}(\boldsymbol{\epsilon}^T\mathbf{S}_\lambda^2\boldsymbol{\epsilon}) + \text{E}\big[\big(\widehat{\beta} - \text{E}(\widehat{\beta}_{\text{pr}})\big)^2\big]n^{-1}\mathbf{x}^T\mathbf{S}_\lambda^2\mathbf{x} \\ &= n^{-1}\sigma^2\text{Tr}(\mathbf{S}_\lambda^2) + \text{Var}(\widehat{\beta}_{\text{pr}})\mathcal{O}(1) \\ &= \mathcal{O}(n^{-1}\lambda^{-d/2m}) + \mathcal{O}(n^{-1}) = \mathcal{O}(n^{-1}\lambda^{-d/2m}) \end{aligned}$$

by Lemma 1.2, Theorem 4.1 (b) and Lemma 1.4 (d). This proves part (b).

The same argument as we used for the partial thin plate spline estimate $\widehat{\mathbf{f}}$ shows that the optimal rate of convergence for minimizing $\text{AMSE}(\widehat{\mathbf{f}}_{\text{pr}})$ is achieved when $\mathcal{O}(\lambda) = \mathcal{O}(n^{-1}\lambda^{-d/2m})$, which leads to $\lambda = \mathcal{O}(n^{-2m/(2m+d)})$ and $\text{AMSE}(\widehat{\mathbf{f}}_{\text{pr}}) = \mathcal{O}(n^{-2m/(2m+d)})$. □

Corollary 4.3. *Suppose $\lambda \approx n^{-\delta}$ for some $0 < \delta < 1$, $f, f^x \in H^m(\Omega)$ are bounded and $m \geq d$. If λ converges at the optimal rate in terms of minimizing $\text{AMSE}(\widehat{\mathbf{f}}_{\text{pr}})$, then*

$$\lambda = o(n^{-1/2}).$$

In particular, the optimal rate for λ ensures that the bias of the partial residual estimate $\widehat{\beta}_{\text{pr}}$ converges faster than the standard deviation of the estimate.

5 Web Appendix E: Derivations for unsmoothed models

In this section we consider in more detail the models we compare in Section 4 of the paper when no smoothing penalty is applied (i.e. $\lambda = \lambda_x = 0$) and include some derivations that help explain our simulation results for these models. In the unsmoothed case, the models are ordinary linear models and the estimated covariate effect $\widehat{\beta}$ and fitted values can be found using simple linear algebra. To avoid confusion, rather than using the same notation for the estimated covariate effect, we denote the estimate by $\widehat{\beta}_{\text{null}}$, $\widehat{\beta}$, $\widehat{\beta}_{\text{RSR}}$, $\widehat{\beta}_{\text{gSEM}}$, $\widehat{\beta}^{+}$ in the null, spatial, RSR, gSEM and spatial+ models, respectively.

Recall that, by the data generation process, each replicate of the response data in the simulation is of the form

$$\mathbf{y} = \beta\mathbf{x} + \mathbf{f} + \boldsymbol{\epsilon}^y$$

where β and $\mathbf{f} = -\mathbf{z} - \mathbf{z}'$ are the true covariate and spatial effects, respectively, and $\boldsymbol{\epsilon}^y$ is iid noise.

In the null and RSR models the estimated covariate effect is the ordinary least squares estimate, in particular, for any given data replicate, the estimate in these models are identical. More specifically,

$$\begin{aligned} \widehat{\beta}_{\text{null}} = \widehat{\beta}_{\text{RSR}} &= (\mathbf{x}^T\mathbf{x})^{-1}\mathbf{x}^T\mathbf{y} \\ &= (\mathbf{x}^T\mathbf{x})^{-1}\mathbf{x}^T(\beta\mathbf{x} + \mathbf{f} + \boldsymbol{\epsilon}^y), \end{aligned}$$

and hence

$$\text{E}(\widehat{\beta}_{\text{null}}) = \text{E}(\widehat{\beta}_{\text{RSR}}) = \beta + \text{E}((\mathbf{x}^T\mathbf{x})^{-1}\mathbf{x}^T\mathbf{f}).$$

So the bias in the null and RSR models is given by $\text{E}((\mathbf{x}^T\mathbf{x})^{-1}\mathbf{x}^T\mathbf{f})$ and is, therefore, directly related to the correlation between the covariate $\mathbf{x}$ and the true unmeasured spatial effect $\mathbf{f}$. Since in our simulations the correlation is negative, the bias in our results is therefore negative. While the covariate effect estimates in the null and RSR models agree, the fitted values differ as the larger model matrix in RSR explains a part of $\mathbf{y}$ that is treated as random noise in the null model. In fact, the column space of the model matrix of the RSR model is the same as that of the spatial model and, therefore, (when $\lambda = 0$) the fitted values in these models agree (i.e. for any given data replicate, the fitted values will be identical).

When no smoothing penalty is applied, the spatial model, the gSEM and the spatial+ model are essentially the same, i.e. for any given data replicate they have the same fitted values and the same unbiased estimate for the covariate effect. The spatial model is an ordinary linear model where the columns in the model matrix are the covariate $\mathbf{x}$ and the spatial basis vectors $\mathbf{B}_{\text{sp}}$. The spatial+ model is a reparametrization of the spatial model where the column $\mathbf{x}$ in the model matrix is replaced by the spatial residuals $\mathbf{r}^x = \mathbf{x} - \widehat{\mathbf{f}}^x$ (where $\widehat{\mathbf{f}}^x$ are the fitted values of a spatial thin plate spline fitted to $\mathbf{x}$). This does not change the overall column space as the difference $\widehat{\mathbf{f}}^x$ lies in the column space of $\mathbf{B}_{\text{sp}}$. By the data generation process,

$$\begin{aligned} \mathbf{y} &= \beta\mathbf{x} + \mathbf{f} + \boldsymbol{\epsilon}^y \\ &= \beta\mathbf{r}^x + \beta\widehat{\mathbf{f}}^x - \mathbf{z} - \mathbf{z}' + \boldsymbol{\epsilon}^y, \end{aligned}$$

with $\beta\widehat{\mathbf{f}}^x - \mathbf{z} - \mathbf{z}'$ in the column space of $\mathbf{B}_{\text{sp}}$ and, therefore, the true effect of $\mathbf{r}^x$ is the same as that of $\mathbf{x}$. In fact, since $\mathbf{r}^x$ is orthogonal to the spatial basis vectors, the estimated effect $\widehat{\beta}^+$ in the spatial+ model (14) of the paper (and therefore $\widehat{\beta}$ in the spatial model (10) of the paper) is obtained as

$$\begin{aligned} \widehat{\beta} = \widehat{\beta}^+ &= (\mathbf{r}^{xT}\mathbf{r}^x)^{-1}\mathbf{r}^{xT}\mathbf{y} \\ &= \beta + (\mathbf{r}^{xT}\mathbf{r}^x)^{-1}\mathbf{r}^{xT}\boldsymbol{\epsilon}^y. \end{aligned}$$

Similarly, for the unsmoothed gSEM, since

$$\mathbf{r}^y = \mathbf{y} - \widehat{\mathbf{f}}^y = \beta\mathbf{r}^x + \beta\widehat{\mathbf{f}}^x - \mathbf{z} - \mathbf{z}' - \widehat{\mathbf{f}}^y + \boldsymbol{\epsilon}^y,$$

with $\beta\widehat{\mathbf{f}}^x - \mathbf{z} - \mathbf{z}' - \widehat{\mathbf{f}}^y$ in the column space of $\mathbf{B}_{\text{sp}}$, the estimated effect of $\mathbf{r}^x$ in the gSEM model (13) of the paper is given by

$$\begin{aligned} \widehat{\beta}_{\text{gSEM}} &= \left(\mathbf{r}^{xT}\mathbf{r}^x\right)^{-1}\mathbf{r}^{xT}\mathbf{r}^y \\ &= \beta + (\mathbf{r}^{xT}\mathbf{r}^x)^{-1}\mathbf{r}^{xT}\boldsymbol{\epsilon}^y. \end{aligned}$$

This shows that $\widehat{\beta} = \widehat{\beta}^+ = \widehat{\beta}_{\text{gSEM}}$. Note that, since $\mathbf{r}^x$ and $\boldsymbol{\epsilon}^y$ are independent

$$\text{E}(\widehat{\beta}) = \text{E}(\widehat{\beta}^+) = \text{E}(\widehat{\beta}_{\text{gSEM}}) = \beta,$$

i.e. the estimated covariate effect is unbiased.

6 Web Appendix F: Additional simulation results

6.1 Mis-specified model

In the simulations of Section 4 of the paper, the data was generated in such a way that the true spatial dependence was that of a thin plate spline. This was ensured by replacing the spatial fields $\mathbf{z}$ and $\mathbf{z}'$ by the fitted values of a thin plate spline model fitted to them. However, in practice, this assumption may not hold and we therefore repeated the simulations for data generated in the same way but where, instead of fitting a thin plate spline model to $\mathbf{z}$ and $\mathbf{z}'$, we used Gaussian process smooths. More specifically, $\mathbf{z}$ has an exponential covariance structure with range parameter 5 and $\mathbf{z}'$ a spherical covariance structure with range parameter 1. Figure 1 shows the results of these simulations. We see that the results are very similar to the simulation results in the paper.

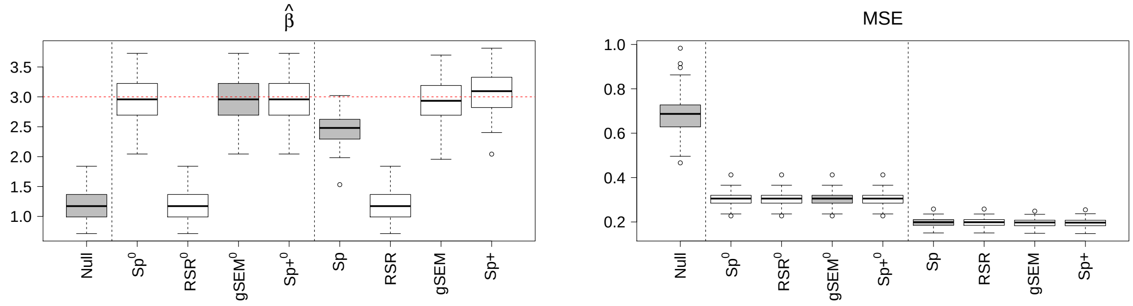

Figure 1: Results of simulations where the true spatial fields $\mathbf{z}$ and $\mathbf{z}'$ are Gaussian process smooths.

6.2 Moderate sample size

In order to investigate the behaviour at moderate sample sizes, we repeated the simulations of Section 4 of the paper for sample sizes $n = 300$, $n = 150$ and $n = 50$ (with spatial basis sizes $k_{\text{sp}} = 100$, $k_{\text{sp}} = 100$ and $k_{\text{sp}} = 30$, respectively). The results shown in Figure 2.

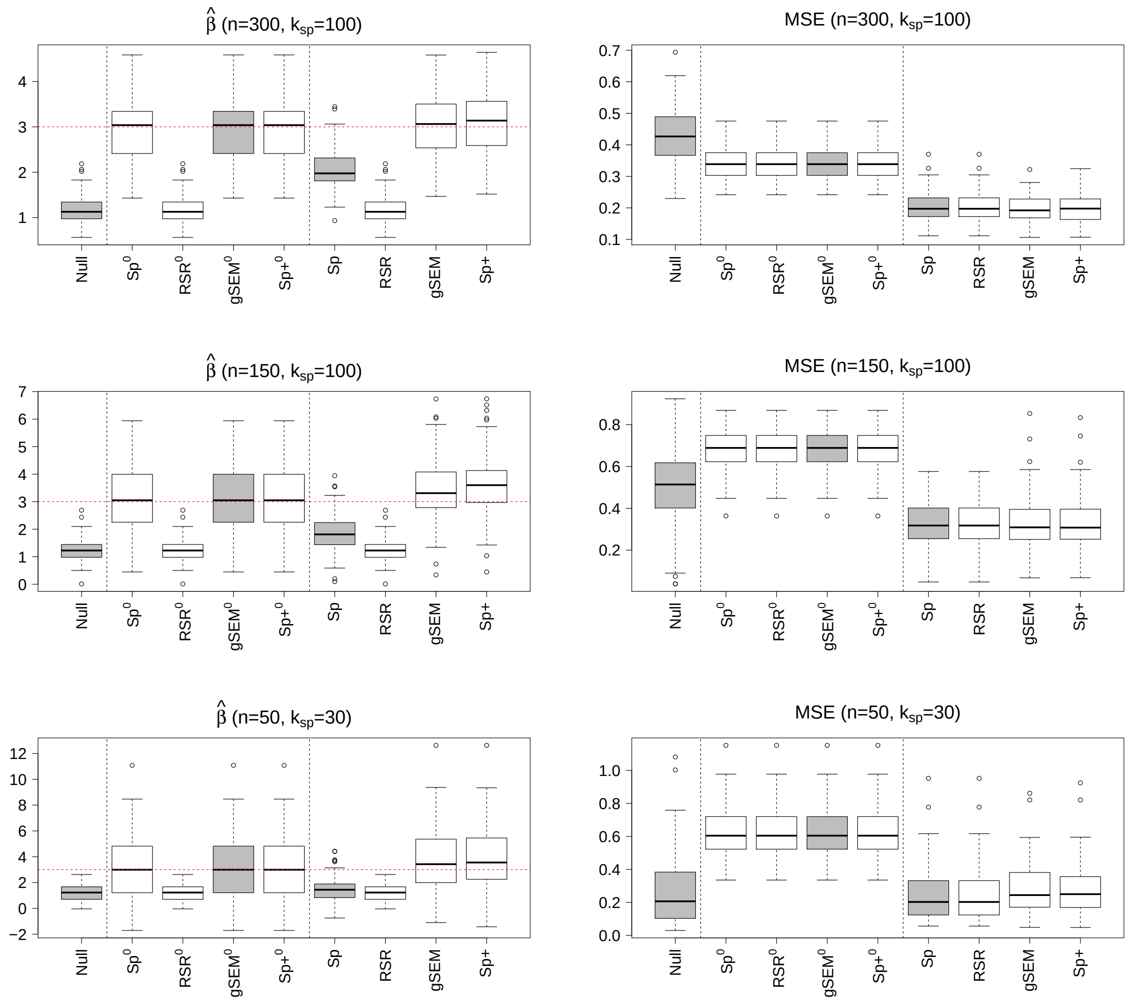

Figure 2: Results of simulations with smaller sample size n.

7 Web Appendix G: Non-Gaussian response data

A distribution is in the exponential family of distributions if its probability density function p can be written in the form

$$p(y) = \exp\left[\{y\theta - b(\theta)\}/a(\phi) + c(y, \phi)\right]$$

where θ and ϕ are parameters of the distribution and a, b and c are functions. This family includes a large number of commonly used distributions in applied statistics, e.g. Gaussian, Poisson, gamma and binomial.

7.1 Spatial model

Suppose we have response data $\mathbf{y} = (y_1, \dots, y_n)^T$ where each y_i is assumed to be a random variable whose distribution is from the exponential family with $\mathrm{E}(y_i) = \mu_i$, and suppose $\mathbf{x} = (x_1, \dots, x_n)^T$ and $\mathbf{t}_1, \dots, \mathbf{t}_n$ are covariate observations and spatial locations as before. A generalized version of (1) in the paper can then be formulated as

$$g(\mu_i) = \beta x_i + f(\mathbf{t}_i) \tag{5}$$

where β is an unknown parameter, f a thin plate spline and $g : \mathbb{R} \to \mathbb{R}$ a link function (i.e. a monotonic smooth function which ensures $g(\mu_i)$ is in the domain of the response variable). The partial thin plate spline estimates of β and $\mathbf{f} = (f(\mathbf{t}_1), \dots, f(\mathbf{t}_n))^T$ are found using a penalized iterative re-weighted least squares (PIRLS) algorithm. Initializing the algorithm with $\widehat{\mu}_i = y_i$ and $\widehat{\eta}_i = g(\widehat{\mu}_i)$, we define so-called pseudodata as $z_i = g'(\widehat{\mu}_i)(y_i - \widehat{\mu}_i) + \widehat{\eta}_i$ and iterative weights $w_i = 1/(g'(\widehat{\mu}_i)^2 V(\widehat{\mu}_i))$ where $V(\mu_i) = \mathrm{Var}(y_i)\phi = b_i''(\theta)a_i(\phi)/\phi$ is the variance function for the distribution of y_i. Let $\widehat{\beta}$ and $\widehat{\mathbf{f}}$ be the minimizers of

$$\|\sqrt{\mathbf{W}}(\mathbf{z} - \beta\mathbf{x} - \mathbf{f})\|^2 + n\phi\lambda\mathbf{f}^T\boldsymbol{\Gamma}\mathbf{f} \tag{6}$$

where $\mathbf{W} = \mathrm{diag}(w_1, \dots, w_n)$ is the weights matrix, $\mathbf{z} = (z_1, \dots, z_n)^T$, and $\lambda > 0$ and $\boldsymbol{\Gamma}$ are as in (2) of the paper. Now redefining $\widehat{\eta}_i = \widehat{\beta}\mathbf{x} + \widehat{\mathbf{f}}$ and $\widehat{\mu}_i = g^{-1}(\widehat{\eta}_i)$, the algorithm is iterated until convergence and the partial thin plate spline estimates $\widehat{\beta}$ and $\widehat{\mathbf{f}}$ are then the minimizers of (6) in the final iteration. Note that, if no smoothing is applied, $\widehat{\beta}$ and $\widehat{\mathbf{f}}$ are the maximum likelihood estimates in a generalized linear model (GLM), which are asymptotically unbiased.

7.2 Spatial+ model

Starting with the model (5), let $\mathbf{W}$ and $\mathbf{z}$ denote the weights matrix and pseudodata at convergence of the PIRLS algorithm. We then define the corresponding spatial+ model as follows. Let $\widehat{\mathbf{f}}^x$ and $\mathbf{r}^x = \mathbf{x} - \widehat{\mathbf{f}}^x = (r_1^x, \dots, r_n^x)^T$ denote the fitted values and residuals in the weighted version of the thin plate regression (4) of the paper with weights $\mathbf{W}$, i.e. $\widehat{\mathbf{f}}^x$ is the minimizer of

$$\|\sqrt{\mathbf{W}}(\mathbf{x} - \mathbf{f}^x)\|^2 + n\lambda_x \mathbf{f}^{xT}\boldsymbol{\Gamma}\mathbf{f}^x$$

with smoothing parameter $\lambda_x > 0$ and $\boldsymbol{\Gamma}$ defined as before. The spatial+ model is then the partial thin plate spline model defined by

$$g(\mu_i) = \beta r_i^x + f^+(\mathbf{t}_i) \tag{7}$$

where β and f^+ are estimated as described in Section 7.1 above. From Section 7.1 we see that the estimates $\widehat{\beta}$ and $\widehat{\mathbf{f}}$ in the spatial model (5) are obtained as the minimizers of (2) in the paper if we replace $\mathbf{y}, \mathbf{x}, \mathbf{f}, \boldsymbol{\Gamma}$ and λ by $\widetilde{\mathbf{y}} = \sqrt{\mathbf{W}}\mathbf{z}$, $\widetilde{\mathbf{x}} = \sqrt{\mathbf{W}}\mathbf{x}$, $\widetilde{\mathbf{f}} = \sqrt{\mathbf{W}}\mathbf{f}$, $\widetilde{\boldsymbol{\Gamma}} = \sqrt{\mathbf{W}}^{-1}\boldsymbol{\Gamma}\sqrt{\mathbf{W}}^{-1}$ and $\widetilde{\lambda} = \phi\lambda$. Thus, at convergence of the PIRLS algorithm, estimation corresponds to that of a Gaussian model for which the model matrix has columns $\widetilde{\mathbf{x}}$ and $\sqrt{\mathbf{W}}\mathbf{B}_{\mathrm{sp}}$. From our comment at the beginning of Section 4.4 of the paper, the decorrelation trick that we used in Section 2.2 of the paper would therefore work if we replace $\widetilde{\mathbf{x}}$ by $\widetilde{\mathbf{r}}$, obtained from a decomposition $\widetilde{\mathbf{x}} = \widetilde{\mathbf{v}} + \widetilde{\mathbf{r}}$ in which $\widetilde{\mathbf{v}}$ is in the column space of $\sqrt{\mathbf{W}}\mathbf{B}_{\mathrm{sp}}$ and $\widetilde{\mathbf{r}}$ is broadly orthogonal to the columns of $\sqrt{\mathbf{W}}\mathbf{B}_{\mathrm{sp}}$. By the properties of weighted thin plate spline regressions, $\sqrt{\mathbf{W}}\mathbf{r}^x$ is broadly orthogonal to $\sqrt{\mathbf{W}}\mathbf{B}_{\mathrm{sp}}$. Therefore, letting $\widetilde{\mathbf{v}} = \sqrt{\mathbf{W}}\mathbf{f}^x$ and $\widetilde{\mathbf{r}} = \sqrt{\mathbf{W}}\mathbf{r}^x$, the required decorrelation is achieved. Finally, replacing $\widetilde{\mathbf{x}}$ by $\widetilde{\mathbf{r}}$ is equivalent to replacing $\mathbf{x}$ by $\mathbf{r}^x$ in the spatial model, leading to the model (7).

7.3 RSR

Recall that in the Gaussian version of RSR, correlation between the covariate and spatial effect estimates is eliminated by restricting the spatial effect to the orthogonal complement of $\mathbf{x}$. In Section 7.2 we saw that estimation in the generalized version of the spatial model (7) corresponds to that of a Gaussian model in which the model matrix has columns $\widetilde{\mathbf{x}} = \sqrt{\mathbf{W}}\mathbf{x}$ and $\sqrt{\mathbf{W}}\mathbf{B}_{\mathrm{sp}}$ with $\mathbf{W}$ the weights matrix at convergence of

the PIRLS algorithm. We can therefore define the generalized RSR model to be the same as the generalized spatial model but with the spatial basis vectors $\mathbf{B}_{\text{sp}}$ in the model matrix replaced by

$$\widetilde{\mathbf{B}}_{\text{sp}} = (\mathbf{I} - \mathbf{x}(\mathbf{x}^T\mathbf{W}\mathbf{x})^{-1}\mathbf{x}^T\mathbf{W})\mathbf{B}_{\text{sp}}.$$

Then, by construction, the generalized RSR model corresponds to a Gaussian model for which the columns $\widetilde{\mathbf{x}} = \sqrt{\mathbf{W}}\mathbf{x}$ and $\sqrt{\mathbf{W}}\widetilde{\mathbf{B}}_{\text{sp}}$ are orthogonal:

$$\widetilde{\mathbf{x}}^T\sqrt{\mathbf{W}}\widetilde{\mathbf{B}}_{\text{sp}} = \mathbf{x}^T\mathbf{W}\widetilde{\mathbf{B}}_{\text{sp}} = \mathbf{0}.$$

References

Chen, H. and Shiau, J.-J. H. (1991). A two-stage spline smoothing method for partially linear models. *Journal of Statistical Planning and Inference* **27,** 187–201.

Rice, J. (1986). Convergence rates for partially splined models. *Statistics & probability letters* **4,** 203–208.

Speckman, P. (1988). Kernel smoothing in partial linear models. *Journal of the Royal Statistical Society: Series B (Methodological)* **50,** 413–436.

Thaden, H. and Kneib, T. (2018). Structural equation models for dealing with spatial confounding. *The American Statistician* **72,** 239–252.

Utreras, F. I. (1988). Convergence rates for multivariate smoothing spline functions. *Journal of approximation theory* **52,** 1–27.
